# Family-based exome sequencing and case-control analysis implicate *CEP41* as an ASD gene

**DOI:** 10.1038/s41398-018-0343-z

**Published:** 2019-01-15

**Authors:** Ashok Patowary, So Yeon Won, Shin Ji Oh, Ryan R Nesbitt, Marilyn Archer, Debbie Nickerson, Wendy H. Raskind, Raphael Bernier, Ji Eun Lee, Zoran Brkanac

**Affiliations:** 1 Department of Psychiatry and Behavioral Sciences, University of Washington, Seattle, WA USA; 2 Department of Health Sciences and Technology, SAIHST, Sungkyunkwan University, Seoul, Korea; 3 Department of Genome Sciences, University of Washington, Seattle, WA USA; 4 Department of Medicine, Division of Medical Genetics, University of Washington, Seattle, WA USA; 5 Division of Medical Science Research, Samsung Medical Center, Seoul, Korea

## Abstract

Autism Spectrum Disorder (ASD) is a complex neurodevelopmental disorder with a strong genetic component. Although next-generation sequencing (NGS) technologies have been successfully applied to gene identification in de novo ASD, the genetic architecture of familial ASD remains largely unexplored. Our approach, which leverages the high specificity and sensitivity of NGS technology, has focused on rare variants in familial autism. We used NGS exome sequencing in 26 families with distantly related affected individuals to identify genes with private gene disrupting and missense variants of interest (VOI). We found that the genes carrying VOIs were enriched for biological processes related to cell projection organization and neuron development, which is consistent with the neurodevelopmental hypothesis of ASD. For a subset of genes carrying VOIs, we then used targeted NGS sequencing and gene-based variant burden case-control analysis to test for association with ASD. Missense variants in one gene, *CEP41*, associated significantly with ASD (*p* = 6.185^e−05^). Homozygous gene-disrupting variants in *CEP41* were initially found to be responsible for recessive Joubert syndrome. Using a zebrafish model, we evaluated the mechanism by which the *CEP41* variants might contribute to ASD. We found that *CEP41* missense variants affect development of the axonal tract, cranial neural crest migration and social behavior phenotype. Our work demonstrates the involvement of *CEP41* heterozygous missense variants in ASD and that biological processes involved in cell projection organization and neuron development are enriched in ASD families we have studied.

## Introduction

Autism Spectrum Disorders (ASD) comprise a heterogeneous group of complex neurodevelopmental conditions diagnosed based on the presence of deficits in social communication and interactions along with restricted, repetitive behaviors, interests or activities (DSM5)^1^. Family and twin studies have provided strong evidence for inherited genetic factors in the etiology of ASD^2,3^. Despite high heritability, the genetic architecture of ASD remains elusive. Advances in array and later sequencing technologies accelerated elucidation of the contribution of de novo copy number variations (CNVs) and de novo single-nucleotide mutations to the molecular basis of sporadic ASD and have estimated that there are hundreds to thousands of ASD risk loci^4–9^. Genomic studies of sporadic autism have also paved the way for using systems biology approaches, such as pathway enrichment and gene co-expression profiling, to implicate neuronal signaling, synaptic transmission, chromatin biology, transcription regulation, cellular proliferation, projection and motility and GTPase/Ras signaling in the etiology of ASD^10–13^.

The frequent detection of de novo variants in ASD supports the role for rare variants in ASD but, in contrast to the studies of sporadic autism, in familial ASD gene discovery has not been as productive^14^. Progress has been made in identification of genes with recessive inheritance in ASD due to power of homozygosity mapping in consanguineous families^15–17^. Studies of individual ASD families, where pedigrees were compatible with Mendelian dominant inheritance, have demonstrated the suitability of exome sequencing to identify candidate ASD variants^18–20^. The medium scale studies that explored the role of dominant inheritance in between 7 and 40 families have identified large numbers of candidate genes and consequently have not followed up with case-control analyses to determine the significance of such findings^21–23^. The two large studies used whole-genome sequencing (WGS) in 85 and 878 families for ASD gene identification^9,24^. The WGS studies utilized numerous analytical approaches including non-coding DNA, exome, CNV, single-nucleotide variant, de-novo, recessive and dominant inheritance analysis. These studies confirmed substantial genetic heterogeneity in familial autism. They ultimately lacked statistical power to identify novel ASD genes as it is estimated that sample sizes to obtain statistical power in exome studies are similar to genome-wide association studies (GWAS) and will probably require more than 25,000 cases^25^. We have previously shown that the study design which utilizes ASD families with distantly related cases (i.e. second and third cousin families) allows for identification of a small number of candidate genes that can be evaluated in case control study to identify association with ASD^26^.

In our current study, we focused on 26 families with at least one affected cousin pair to identify private gene disrupting and missense variants of interest (VOI) in each family. The genes we identified are enriched for “cell projection organization” and “neuron development”, which are categories we found enriched in known ASD genes as well. Furthermore, for a subset of candidate genes we performed a gene-based case-control analysis in a larger sample and found significant association with *CEP41*, which we further confirmed using the family-based transmission disequilibrium test (TDT). We then used a zebrafish model to characterize the functional effects of the *CEP41* variants identified in the case-control study. The zebrafish studies showed that the expression of the variants in embryos induces axonal defects and also affects cranial neural crest (CNC) cell migration. Furthermore, zebrafish carrying the *CEP41* variants we identified in ASD cases exhibited deficits in social behavior.

In summary, our familial exome sequencing analysis identified novel ASD candidate genes that are enriched for neuronal development and cell projection organization. Case-control analyses of a subset of candidate genes revealed a significant ASD association with *CEP41*, which was confirmed by TDT. Furthermore, we showed in a zebrafish model that ASD-associated *CEP41* variants affect axonal development, CNC migration and social behavior.

## Methods

### Study sample

For the family-based exome sequencing study, we identified 26 ASD families with affected first cousins from the NIMH repository (https://www.nimhgenetics.org/). For each family, we obtained DNA from the affected cousins and their affected siblings when available, for a total of 61 affected subjects. The pedigrees for the families used in the study are shown in Supplementary Information.

For case control studies, we obtained 1143 familial Caucasian cases and 1168 Caucasian controls as described previously^26^. In brief, the subjects were obtained from the NIMH repository, a University of Washington (UW) multiplex autism collection and a UW study on dyslexia. For both NIMH and UW ASD subjects, the diagnostic status was determined by gold-standard ADOS and ADI-R assessment followed by expert clinical judgment using all available information. The NIMH control sample consists of adults who completed an online short self-report assessment to exclude severe psychiatric disorders, but were not specifically screened for autism. The UW controls were ascertained for dyslexia by comprehensive testing, and screened for absence of ASD with a questionnaire. All samples from the NIMH collection and UW were utilized in concordance with Institutional Review Board approvals from all participating institutions for sample and data sharing.

### Whole-exome sequencing, variant calling, annotation and prioritization

Nimblegen SeqCap EZ Human Exome Library v2.0 (Roche, Basel, Switzerland) was used to capture the exomes, followed by paired-end 50 bp sequencing on an Illumina HiSeq2000 sequencing platform. Sequences were generated in the UW Genome Sciences Center for Mendelian Genomics^27^. Raw sequencing files were processed for quality control with FastQC (http://www.bioinformatics.babraham.ac.uk/projects/fastqc/). Exome-captured sequencing reads were aligned to NCBI human reference genome GRCh37 (hg19) using Burrows–Wheeler Aligner (BWA v0.7.10)^28^. Aligned reads were subsequently processed using Picard v1.118 (http://picard.sourceforge.net) for duplicate removal. Thereafter, Genome Analysis Toolkit (GATK v3.2) was used for variant calling^29^. ANNOVAR^30^ (v2014) was used for annotating the single-nucleotide variants called by GATK.

To identify VOIs we used multistep filtering. Initial filtering included the removal of intergenic and 3′/5′ UTR variants, non–splice related intronic variants and synonymous variants. Variants were further filtered based on 1000 Genomes (1KG) “European”, Exome Sequencing Project (ESP) European American and dbSNP databases. For the most stringent frequency filtering, we retained only “private” variants, which we define as variants not present in any of the data sets (dbSNP132, 1KG (2012 April) and ESP). Finally, to be marked as a VOI, the variant needed to be present in all affected individuals in a family. Predictions on variant deleteriousness based on SIFT, Poly-Phen-2 and GERP scores were obtained through ANNOVAR (v2014). Taking into consideration bioinformatics predictions of variant deleteriousness, to select genes for case-control association studies, we performed manual PubMed searches and evaluated genes based on available information on brain expression and function.

### Targeted gene sequencing, variant calling and annotation

We employed molecular inversion probes (MIPs) for targeted capture of the coding region of the selected genes^31^. We designed MIPs for protein-coding regions of 13 genes and performed sequencing and analysis as described previously (details in Supplementary Information). Sequence reads were aligned to NCBI human reference genome GRCh37 (hg19) using Burrows–Wheeler Aligner (BWA v0.7.10)^28^. Variants were called with GATK^29^ (v3.2) unified genotyper. Thereafter, ANNOVAR^30^ (v2015) was used to annotate the single-nucleotide variants as nonsynonymous, splice, stop gain, stop lost or synonymous variants. For MIP sequencing analysis, we used slightly less stringent criteria to identify “rare” variants, which we defined as variants with frequency <0.01 in 1KG European samples. For the case-control association analysis, we used rare protein altering variants (nonsynonymous, splice, stop gain, stop lost).

### Variant validation and evaluation in family members

Variants in the genes selected for case-control analysis and rare *CEP41* variants identified in cases and controls were confirmed by Sanger sequencing as previously described^23^. For cases with *CEP41* variants, available affected siblings were evaluated for presence of the variant allele using Sanger sequencing.

### Association analysis

For the case-control study, we performed gene-based variant burden association analysis^32^. For each gene, we aggregated all rare protein-altering variants. Significance of the burden association was determined with one-sided Fisher exact test.

For *CEP41*, we performed TDT analyses^33^ to further evaluate the association with ASD. TDT uses *Χ*^2^ goodness of fit statistics to determine if there is a preferential transmission of a risk allele to the affected cases. In the TDT analysis, we used affected siblings of cases with rare protein-altering variants in *CEP41*, under the assumption that if *CEP41* variants contribute to the phenotype, we should see over transmission of risk variants to affected siblings. This analysis incorporated a rare-variant extension of the TDT. We used a TDT-burden of rare variants that counts the number of minor-allele-transmission events to affected siblings.

### Functional enrichment analysis

Genes with VOIs identified in exome sequencing were subjected to WebGestalt^34^ for functional enrichment analysis using the Overrepresentation Enrichment Analysis (ORA). We interrogated the Gene Ontology (GO) database for enrichment in three categories: “Biological Process”, “Cellular Component” and “Molecular Function”. The focus of enrichment analysis was to identify broad biological processes that are involved in ASD. As the current estimates for the number of genes in ASD ranges from hundreds to thousands^4–9^, to reflect ASD number of genes estimates, we set the parameters used in the analysis to Minimum Number of Genes = 500 and Maximum Number of Genes = 1500. False Discovery Rate (FDR) analysis was performed by Benjamini–Hochberg procedure as a measure of control for false positives. Enrichment was considered significant for FDR below 0.05.

For enrichment analysis of known ASD genes, we used the SFARI gene^35^ database (https://gene.sfari.org/). In our analysis, we included SFARI genes for syndromic disorders that have ASD as part of the phenotype (Category S) and genes that are identified as having the strongest evidence for involvement based on rigorous statistical case-control comparisons (Categories 1 and 2). For SFARI ASD gene enrichment analysis, we used the same analysis parameters as for VOI gene analysis.

### DNA plasmid construction

Human wild-type (WT) *CEP41*, purchased from Dharmacon, was mutated to generate variants found in the case-control study using QuickChange II Site-Directed Mutagenesis Kit (Agilent, #200524, USA). Zebrafish WT *cep41* was amplified with cDNAs obtained from 3 days post-fertilization (dpf) zebrafish and mutated to generate MO-insensitive construct. The human and zebrafish *CEP41* plasmid constructs were subcloned into the pCS2+ vector for expression in zebrafish.

### Zebrafish housing and manipulations

Adult WT zebrafish (AB strain) were maintained with a cycle of 13 h light and 11 h dark in the automatic system (Genomic-Design, Korea) at 28.5 °C and pH 7.0–7.9. The zebrafish embryos were collected by natural breeding and incubated in clean Petri dishes containing E3 medium (297.7 mM NaCl, 10.7 mM KCl, 26.1 mM CaCl_2_ and 24.1 mM MgCl_2_) containing 1% methylene blue (Samchun chemicals, M2662, Korea) at 28.5 °C. To inhibit the formation of melanin, which interferes with immunostaining, the zebrafish larvae were raised in E3 medium containing 0.2 mM *N*-phenylthiourea (PTU; Sigma-Aldrich, p7629, USA).

### Microinjection into zebrafish

To block the expression of zebrafish *cep41*, translation-blocking antisense morpholino oligonucleotides (MOs) were designed and synthesized by GeneTools (Philomath, OR, USA). The MOs (5′-CATCTTCCAGCAGCAGAGCTTCGGC-3′) were dissolved in nuclease-free water (2.5 µg/µl) and microinjected into zebrafish embryos using a gas-used microinjection system (World Precision Instruments, PV83 Pneumatic PicoPump, SYS-PV830, FL, USA). The efficiency of the designed MOs was verified by Western blot assay for CEP41 using control zebrafish and *cep41* morphants. Capped mRNAs of WT or mutated human *CEP41* variants were synthesized by mMESSAGE mMACHINE kit (Ambion, AM1340, USA). The in vitro synthesized mRNAs (150–300 ng/µl) were injected into zebrafish embryos together with *cep41* MOs.

### Immunohistochemistry (IHC) and imaging of zebrafish embryos

Zebrafish embryos at 6–13 somites stage were fixed in 4% paraformaldehyde (PFA) for 14 h at 4 °C. After two times washing with 1× phosphate buffered saline (PBS), embryos were blocked with blocking solution (10% normal goat serum (NGS) in 1× PBST (0.5% Triton X-100 in 1× PBS)) for 2 h at room temperature. Thirty-five hpf zebrafish embryos were dechorinated and soaked in 90% methanol for 1 min at −20 °C. After washing with 1× PBS, the embryos were fixed in 4% PFA at 4 °C overnight and incubated in equal volume of 2× fixation buffer (0.2 M PBS containing 8% sucrose and 0.3 mM CaCl_2_) for 2 h at 4 °C. The embryos were rinsed with PBS after fixing and their eyes were removed before permeabilization with cold acetone (−20 °C for 20 min) and 10 μg/ml of proteinase K (RT for 10 min). They were rinsed again with 0.1 M PBS three times (for 5 min each). To prevent nonspecific binding of the secondary antibodies, embryos were kept for 1 h at 37 °C in blocking solution (1× PBS, 1% BSA, 1% DMSO and 2% donkey serum). The zebrafish embryos were incubated with mouse anti-acetylated-tubulin antibodies (Sigma, T7451, MO, USA, 1:400) in blocking buffer (1× PBS, 1% BSA, 1% DMSO and 2% donkey serum) or with mouse anti-SOX10 antibodies (Santa Cruz, sc-365692, USA, 1:50) in blocking buffer (1× PBST and 5% NGS) at 4 °C overnight. These zebrafish embryos were then incubated with AlexaFlour®488-conjugated secondary antibodies (Invitrogen, mouse A11001, 1:500) at RT for 2 h after washing with PBST. The immunostained zebrafish embryos were mounted with 75% glycerol for photography using a fluorescence microscope (Nikon, SM21270, Japan) or embedded in 1% low melting agarose (Mentos, M2070, Germany) dissolved in E3 medium for photography using a confocal microscope (Carl Zeiss, LSM700, Germany). The images were analyzed by NIS-Elements software (Nikon, Japan) or Zeiss ZEN imaging software (Carl Zeiss, Germany). For statistical analyses, zebrafish were allocated to experimental groups without randomization and blinding

### Zebrafish behavioral analysis

Based on the established rodent models^36^ and proposed zebrafish models of ASD^37^, we developed a group preference test to evaluate zebrafish social behavior. In a group preference test, we evaluate if the target fish has a preference to spend more time close to a group, thereby indicating a preference for social interactions. We performed this paradigm in adult 4-month-old WT and *cep41+/−* fish and in 5–6 dpf larvae injected with control MO, *cep41* MO and human *CEP41* mRNAs variants.

Four-month-old adult zebrafish (WT and *cep41+/−*) were individually placed into a tank (length 18 × height 7 × width 8 cm) filled with 500 ml of system water. The tank was divided into two sections by placing a transparent plastic plate at the center. A group of six adults (three male and three female) was placed as a social cue in the first section and a target fish was placed in the second section. For measurement, the second section was divided into four zones based on proximity to the group. The target fish location was monitored for 17 min during light-on interval. Zebrafish larvae were individually placed into chambers (7.3 × 1 × 3 cm) filled with 10 ml of E3 media, which were divided into two sections by placing a transparent glass plate at a quarter of length. A group of five larvae were offered as a social cue in the smaller section and the target larva was placed in the larger section, which was again divided into four zones for measurement purposes. The larva’s zone preference during light-on conditions was monitored for 1 h.

All recordings were performed using a Daniovision (Noldus, Wageningen, Netherlands) imaging system. The recorded video images were analyzed to quantify the cumulative time at each zone and measure target fish velocity using Ethovision XT software (Noldus, Wageningen, Netherlands). Five different target zebrafish larvae or six different target zebrafish adults were individually recorded in each analysis that was replicated. For statistical analyses, zebrafish were allocated to experimental groups without randomization and blinding.

## RESULTS

### Exome sequencing and analysis

For 61 sequenced exomes, the average read depth was 64×, with 82% of exome sequence covered at a depth greater than 20× and 93% covered at 8×. Among all 61 samples, 98,430 coding single-nucleotide variants (SNVs) were identified of which 43,850 were non-synonymous. On average, each exome contained approximately 23,500 SNVs, of which ~12,500 were non-synonymous. The coverage statistics for exome sequencing is presented in Supplementary Table 1. Following variant function and frequency filtering, as described in Methods, we identified 0–17 VOIs per family, for a total of 145 VOIs in 139 genes (Supplementary Table 2). For VOIs, 137/145 were present in a single family and four were present in two families. To facilitate the comparison of identified variants with other exome sequencing studies, we evaluated the predicted pathogenicity of the variants with SIFT, Polyhen2, GERP and CADD. Based on literature review which included information on gene expression in brain, gene function, involvement in neurogenetic disorders, and taking into consideration predicted variant pathogenicity scores, we selected 13 genes for the next-stage association study in a larger set of case and control samples: *BAI2, CEP250, CEP41, CEP78, DCDC2, NFATC1, NTAN1, SCN10A, SH3BP4, SNPH, SPATA3, TMEM82* and *ZNF638*. Each of the 13 selected candidate genes was present in a single family only. Sanger sequencing confirmed the VOIs in all 13 candidate genes. All exome sequences reported in this study were deposited in NDAR (https://ndar.nih.gov/edit_collection.html?id=1919). Available phenotype information for the exome-sequenced samples is reported in Supplementary Table 3.

### Functional enrichment analysis

Our hypothesis was that the shared VOIs identified in our exome-sequencing analysis are enriched for the genes that are relevant for brain development, as such genes are more likely to be involved in ASD susceptibility. Biological process analysis identified significant association for the involvement of genes containing VOIs with seven biological processes. These processes fall under two broad categories: “cell projection organization” (GO:0030030, FDR = 0.0039) and “neuron development” (GO:0048666, FDR = 0.0321). The cell projection organization includes processes that result in assembly, arrangement or disassembly of cellular parts such as cilia or axon. The neuronal development GO process includes proteins involved in progression of a neuron in early development from initial commitment of the cell to neuronal fate to fully functional neuron. This includes the anatomical structures of neuron projection such as axons or dendrites. The enrichment analysis for cellular components identified association with seven GO terms including “cell junction” (GO:0030054, FDR = 0.0063), “microtubule cytoskeleton” (GO:0015630, FDR = 0.0098) and “cell surface” (GO:0009986, FDR = 0.0324). The cell junction is a specialized region where anchoring proteins extend through plasma membrane to form connections with neighboring cells or with extracellular matrix, while the microtubules (MTs) and associated proteins form the internal cell scaffolding. Molecular functions identified as enriched in our analysis include proteins involved in “kinase binding” (GO:0019900, FDR = 0.0016). The GO is hierarchically organized and individual genes are included in multiple categories. The enriched genes and individual categories of biological processes, cellular components and molecular functions we identified are consequently overlapping as well. Thus, in Table 1, we presented a subset of enriched categories that contain all the genes with VOIs that were enriched. All categories enriched with FDR < 0.05 are presented in Supplementary Table 4.

**Table 1:** Subset of the overrepresentation enrichment analysis using Gene Ontology (GO) database for the genes with VOIs identified by exome sequencing

GO category	GO term	*C*	*O*	*E*	*R*	*p* Value	FDR	Overlapping genes
Biological process	Cell projection organization (GO:0030030)	1268	23	8.66	2.66	1.26E−05	0.0039	CEP250;FGD5;TTC21A;FSTL4;LHX1;MAP1B;MAP2;EIF2AK4;NCK1;MINK1;TRPV2;DCDC2;RAPGEF6;PLXNB1;CENPJ;PRKCQ;IFT46;LRFN2;KLHL1;BHLHB9;SSH2;SCARF1;CEP41
	Neuron development (GO:0048666)	951	16	6.49	2.46	7.17E−04	0.0321	FSTL4;LHX1;MAP1B;MAP2;EIF2AK4;NCK1;MINK1;TRPV2;PLXNB1;PRKCQ;LRFN2;KLHL1;WNK1;BHLHB9;SSH2;SCARF1
Cellular component	Cytoskeletal part (GO:0044430)	1489	17	6.59	2.58	2.22E−04	0.0063	TUBGCP2;CEP250;NEK10;EPB41;FBXO5;KRTAP19-3;KRT86;MAP1B;MAP2;DCDC2;INO80;TCP11L1;CENPJ;IFT46;CEP78;CEP41;SNPH
	Cell junction (GO:0030054)	1357	16	6.00	2.67	2.45E−04	0.0063	CHRM4;EPB41;FGFR4;MPRIP;ARHGEF16;PCLO;ITGB6;MAP1B;NCK1;MINK1;DUOX2;PLEKHG5;LRFN2;CAT;CEP41;SNPH
	Synapse part (GO:0044456)	626	8	2.77	2.89	6.35E−03	0.0324	CHRM4;EPB41;PCLO;MAP1B;IQSEC3;MINK1;LRFN2;SNPH
Molecular Function	Kinase binding (GO:0019900)	587	13	3.45	3.77	3.81E−05	0.0016	CEP250;FAM83C;EPHA1;WWC1;FBXO5;CNPPD1;ARHGEF16;NCK1;NFATC1;PPP1R12C;CENPJ;WNK1;HYAL2

C number of reference genes in the category, O number of observed genes in the category, E expected number of genes in the category, R ratio of enrichment, *P* value *P* value from hypergeometric test, FDR FDR using Benjamini–Hochberg procedure

The enrichment analysis of 111 SFARI category S, 1 and 2 confirmed that enrichment categories we identified in VOI analysis are enriched in ASD as well. The majority of the categories overrepresented in VOI analysis, including “cell projection organization” and “neuron development”, are also enriched in SFARI genes. All the enriched categories for SFARI genes are presented in Supplementary Table 5.

### Targeted sequencing and variant burden association analysis

To evaluate the statistical evidence for association of 13 selected candidate genes with ASD, we performed gene-based variant burden association analysis. Using MIP enrichment, the coding regions of these 13 genes were sequenced in 1143 cases and 1168 controls. After removal of duplicated and poorly captured samples, 1004 unrelated familial cases (NIMH cases = 800, UW cases = 204) and 1127 controls (NIMH controls = 924, UW controls = 203) were used for burden analysis. MIP sequencing and subsequent variant calling identified 992 variants within the 13 candidate genes. Following the multi-step approach described in the methods that includes variant annotation and frequency filtering, 277 rare protein-altering variants were included in case-control analyses (Supplementary Table 6).

The gene-based variant burden analysis identified a statistically significant association of *CEP41* with ASD (Supplementary Table 7). We observed 18 *CEP41* rare protein-altering variants in cases and two in control samples (*p* = 6.185e−05, OR = 10.26, 95%CI [2.37–44.36]). One variant, p.P206A, was observed in 12 cases and one control (*p* = 8.527e−05, OR = 13.69, 95%CI [1.77–105.48]). In addition, this p.P206A variant is present in the ExAC database at a frequency of 0.003719 in the European population (*p* = 0.0522, OR = 1.62, 95%CI [0.91–2.91]). All the *CEP41* variants identified with MIP-based sequencing were confirmed by Sanger sequencing. The *CEP41* variants are reported in Table 2. It is notable that rare variants identified in cases have bioinformatics scores as determined by SIFT, Polyphen, GERP and CADD that are more consistent with deleterious effects, as compared to a single variant p.A107G that was identified in controls.

**Table 2:** Rare protein altering variants identified in CEP41 in case-control study

Position	Ref/ Alt	AA change	Case Obs	Control Obs	SIFT	Polyphen	GERP	CADD (Phred)
chr7:130038866	C/G	A330P	1	0	0.12	0.06	2.66	7.084
chr7:130040580	C/T	R242H	1	0	0.04	0.998	6.03	34
chr7:130041748	G/C	P206A	12	1	0.14	0.997	5.54	23.3
chr7:130041754	T/C	M204V	1	0	0.15	0.64	5.54	18.04
chr7:130041762	G/C	S201C	1	0	0.03	0.997	5.54	21.9
chr7:130044507	G/C	A107G	0	1	0.39	0.116	0.835	10.79
chr7:130050987	C/G	A85P	1	0	0.28	0.773	3.18	15.08
chr7:130056798	A/G	M36T	1	0	0	0.302	5.71	13.96

SIFT (Sorting Intolerant From Tolerant) predicts impact of amino acid substitutions based on the degree of conservation in sequence alignments derived from closely related sequences. Scores < 0.05 are considered deleterious. PolyPhen-2 (Polymorphism Phenotyping v2) predicts impact of a variant on the structure and function of a human protein using eight sequence-based and three structure-based predictive features. Scores > 0.95 are considered probably damaging. GERP (Genomic Evolutionary Rate Profiling) identifies functional constraint of a sequence variant by quantifying substitution deficits in multiple alignments. Substitution deficits represent a natural measure of constraint that reflects the strength of past purifying selection. Higher GERP scores are more deleterious. CADD (Combined Annotation Dependent Depletion) is a framework that integrates multiple annotations into one metric by contrasting variants that survived natural selection with simulated mutations. Higher scores indicate increased deleteriousness

The TDT was performed on 37 affected siblings of cases that had rare *CEP41* variants. We found over-transmission of rare alleles to affected siblings: 27 rare alleles, 10 WT alleles (*χ*^2^ = 6.9; *p* = 0.008, Yates’ corrected).

### ASD subject phenotype analysis

Available phenotypic information for 54 subjects from 26 exome-sequenced families as well as their pedigrees is provided in Supplementary Information and Supplementary Table 3. For 28 subjects, we had verbal IQ, and for 30 subjects, we had non-verbal IQ information. In this sample, the mean verbal IQ was 85.5 (SD = 26.22) and mean non-verbal IQ was 101.1 (SD = 17.11). Phenotype analysis for subjects with *CEP41* mutations did not reveal any specific phenotype characteristics for available measures. Phenotype information for subjects with *CEP41* mutations is shown in Supplementary Table 8.

### CEP41 variants affect zebrafish axonal tract development

**Fig. 1:**
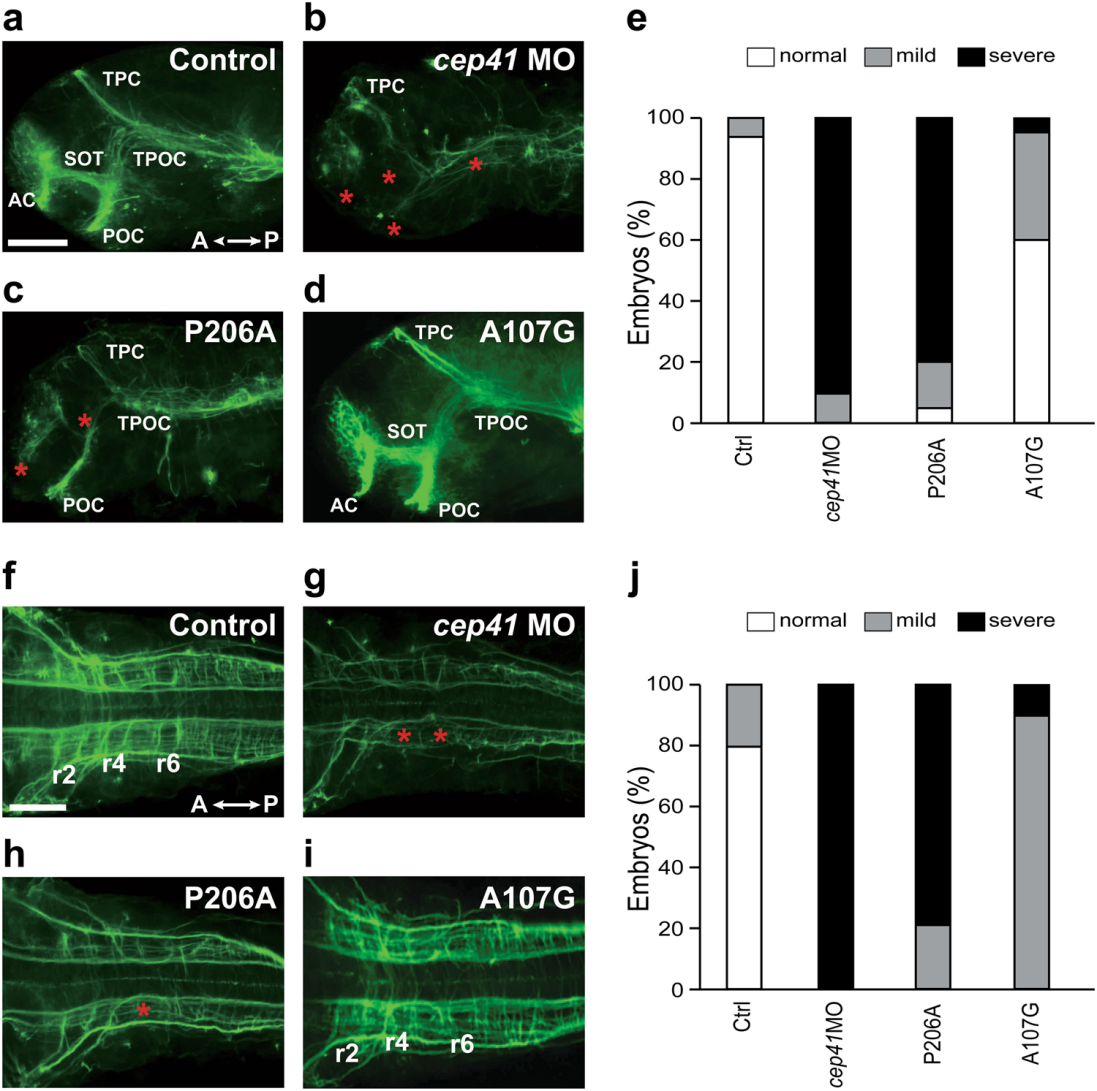
Expression of human *CEP41* variants causes axonal deficits in zebrafish brain. **a**–**d** Axon tracts of the forebrain in zebrafish microinjected with *cep41* MOs or human *CEP41* mRNAs as indicated in each panel. The axons immunostained with anti-acetylated α-Tub antibodies at 35 hpf were imaged by fluorescence microscopy. AC anterior commissures, POC post optic commissure, SOT supra optic tract, TPOC tract of the post optic commissure, TPC tract of the posterior commissure. Asterisks indicate reduced and disorganized axons. Scale bar is 100 µm. **e** Quantified data by counting zebrafish embryos with forebrain axonal defects. The severity of defective axons is distinguished as the numbers of malformed axonal tracts. Mild: 1–2 axonal defects. Severe: 3–5 axonal defects. **f**–**i** Axon tracts of the hindbrain in MOs or mRNA-injected zebrafish. r2, r4 and r6 are rhombomere 2, 4 and 6. Asterisks indicate defective axons and scale bar is 100 µm. **j** Quantified data by counting zebrafish embryos with hindbrain axonal defects. The severity of defected axons is distinguished as the numbers of abnormal axons. Mild: 1–6 axonal defects. Severe: 7–10 axonal defects

On the basis of prior work^38^ showing functional involvement of *CEP41* in MTs that are important for axonal development, we wanted to evaluate the effect on axonal development of *CEP41* variants found in ASD. We analyzed the brain axonal tracts at 35 h post-fertilization (hpf) zebrafish after exogenous expression of the variants by immunostaining with acetylated α-tubulin antibodies. The axonal phenotypes in zebrafish injected with mRNA of human *CEP41* variants were compared to those of zebrafish injected with *cep41* MOs. The knockdown efficiency of the *cep41* MOs was validated by western blotting (Supplementary Figure 1a). Common morphological defects, such as curved tails and heart edema, of the *cep41* morphants and the p.P206A variant-expressing zebrafish were in accordance with previously published results^38^ (Supplementary Figure 1b). The control zebrafish showed intact main white matter tracts, representing axonal bundles in the forebrain (Fig. 1a) and clearly delineated rhombomere segments in the hindbrain (Fig. 1f). In contrast, the *cep41*-deficient zebrafish revealed severe axonal tract impairments in both forebrain (Fig. 1b) and hindbrain (Fig. 1g). More than 80% of *cep41* knockdown zebrafish showed missing or reduced tracts in the anterior commissure (ac), the posterior commissure (pc), and the supra optic tract (sot) of the forebrain and disorganized rhombomere segments in the hindbrain. Remarkably, the zebrafish expressing *CEP41* p.P206A revealed similar axonal defects to those of the *cep41* knockdown zebrafish (Fig. 1c, h), whereas the zebrafish expressing p.A107G variant found in control individuals produced relatively mild axonal defects (Fig. 1d, i). Moreover, the axonal deficits were observed in the zebrafish expressing additional *CEP41* variants identified in ASD cases: p.R242H, p.S201C, p.M30T, p.A85P, p.M204V and p.A330P (Supplementary Figure 2).

We further tested the function of ASD-associated *CEP41* variants in *cep41* knockdown zebrafish in order to demonstrate the specificity of the axonal impairments driven by expression of *CEP41* variants. Exogenously expressed WT *CEP41* largely restored the axonal deficits of *cep41*-deficient zebrafish (Supplementary Figure 3b and 3m). The p.A107G variant identified in controls restored the axonal impairments (Supplementary Figure 3d and 3o), whereas p.P206A (Supplementary Figure 3c and 3n) and six additional variants (Supplementary Figure 3e-j and 3p-u) identified in ASD cases failed to rescue the defects in both forebrain and hindbrain axonal tracts. Taken together, these results suggest that the *CEP41* variants identified in ASD affect axonal tract formation indicating that the role of CEP41 in axonal development may be a mechanism responsible for ASD pathology.

### CEP41 is involved in migration of CNC cells in zebrafish

**Fig. 2:**
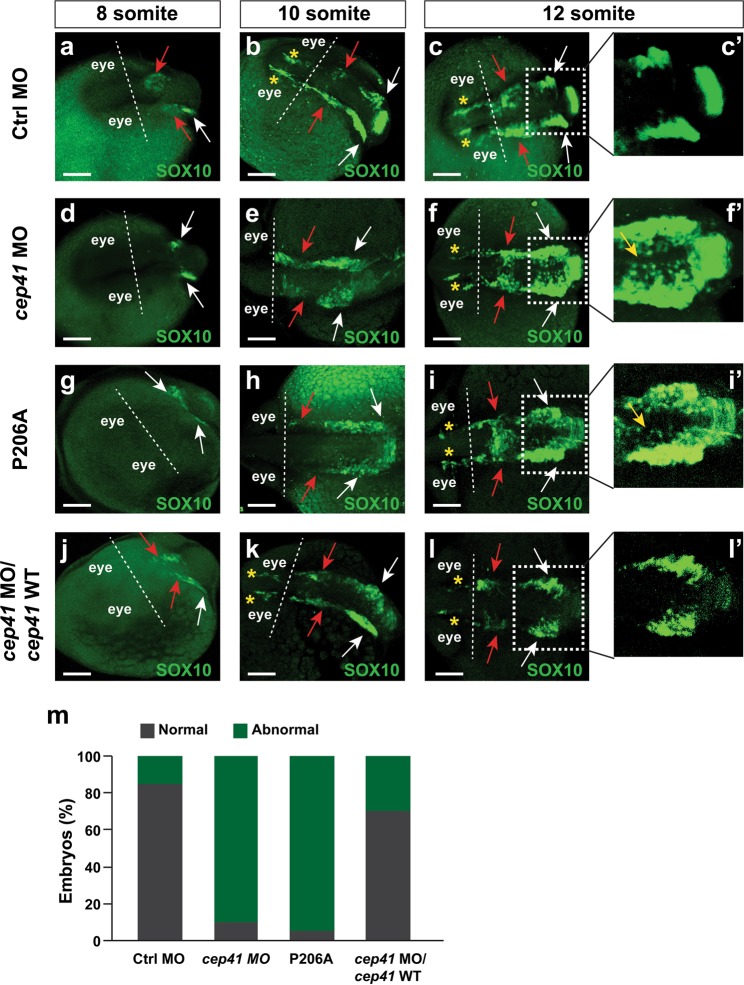
The depletion of *CEP41* affects migration of the cranial neural crest (CNC) cells in zebrafish. Analysis of migration of CNC cells in the control MO (**a**–**c**), *cep41* MO (**d**–**f**), P206A (**g**–**i**) and *cep41* MO+ zebrafish *cep41* mRNA (**j**–**l**)-injected zebrafish at 8, 10 and 12 somite stages. Asterisks, red arrows and white arrows indicate migrating CNC cells in the forebrain, midbrain and hindbrain, respectively. The extent of medial expansion of CNC cells (yellow arrows, zoomed in area) was compared in the hindbrain of each zebrafish (**c′**, **f′**, **i′**, **l′**). **m**. All data are representative of at least five independent experiments. The quantified data of zebrafish with defective CNC cell migration are graphically presented (20 embryos/counting). Abnormal: either delayed migration or medial expansion of CNC cells

In ASD, deficits in neuronal migration were proposed as one of the pathophysiological mechanisms^39,40^ and our functional enrichment analysis identified biological processes related to “neuron development” (GO:0048666) in VOI candidate gene analysis. We used a zebrafish model of CNC cell migration as a proxy to evaluate the effects of *CEP41* variants on neuronal development. Such a model was previously used to evaluate effects of *DISC1*, a gene implicated in brain development and multiple brain disorders including autism^41,42^. The depletion of *cep41* and expression of p.P206A variant caused delayed migration of CNC cells during 8–12 somites stage in zebrafish brain (Fig. 2). The migrated CNC cells were not observed in the midbrain and the forebrain of *cep41*-deficient zebrafish at 8 and 10 somite stages, respectively (Fig. 2d–e, g–h). Similarly to what was observed in *disc1* morphants^42^, the *cep41*-defective zebrafish revealed medial expansion, along with hindered lateral migration of CNC cells at 12 somite stages (Fig. 2f′, i′). The CNC cells successfully migrated in the zebrafish co-injected with *cep41* MOs and MO-insensitive zebrafish *cep41* mRNA (Fig. 2j–l), thereby indicating a specific role of CEP41 in migration of CNC cells. Taken together, these results suggest that CEP41 acts on neural crest cell migration.

### CEP41 variants identified in ASD affect zebrafish social behavior

To determine whether *CEP41* variants affect behaviors that characterize ASD, we examined preference for social interactions of the *cep41*-ablated zebrafish. First, we examined the behavior of 4-month-old WT zebrafish to evaluate our experimental task. For WT fish, we observed that they preferentially spend time in proximity of the group, which is consistent with preference for social interactions (Supplementary Figure 4b). However, 4-month-old *cep41+/−* fish did not show preference for group proximity (Supplementary Figure 4c) indicating that molecular function of CEP41 is associated with zebrafish social behavior.

**Fig. 3:**
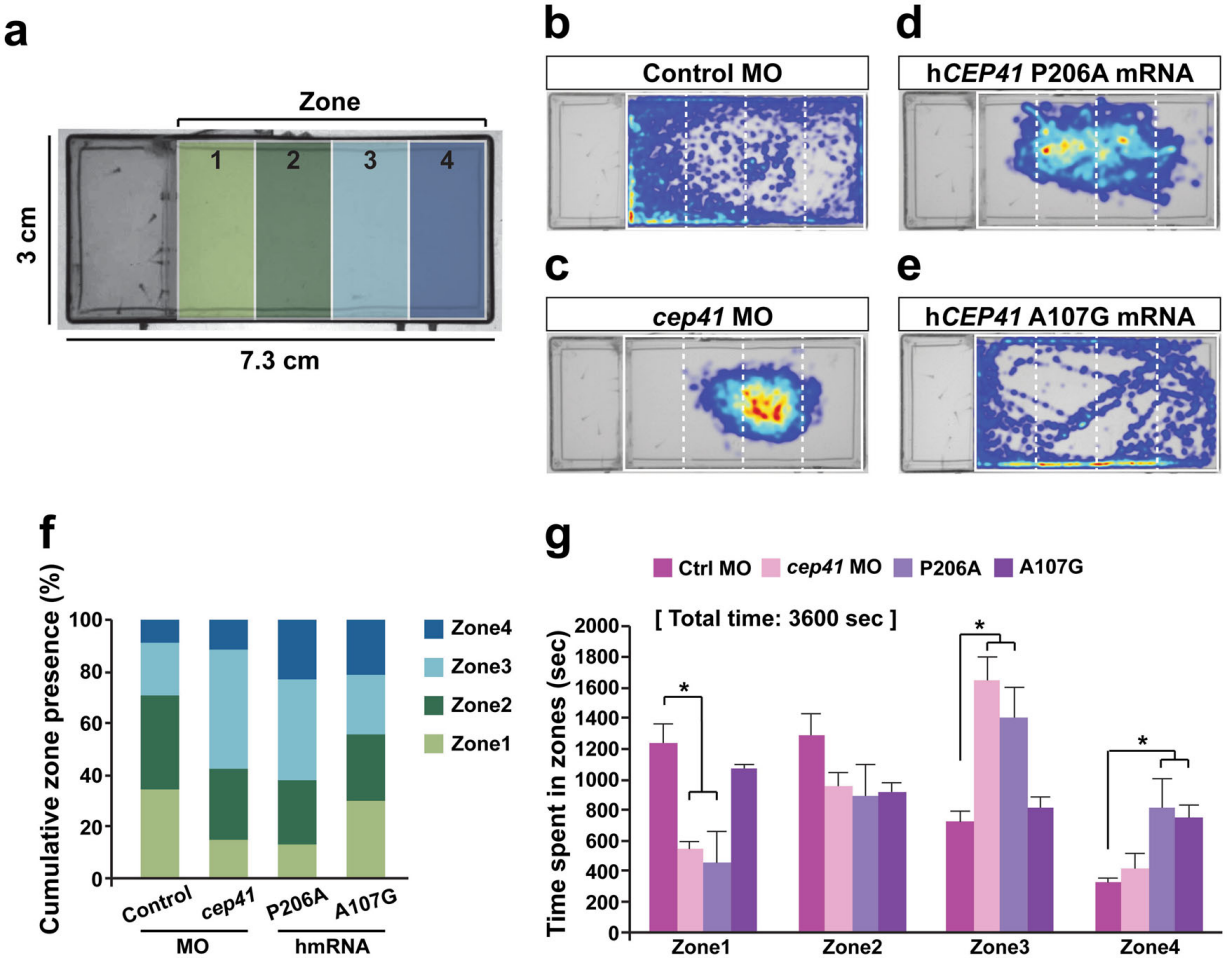
The deficiency of *CEP41* affects social behavior in zebrafish larvae. **a** A diagram of experimental chamber indicating the zones for the analysis of target location. **b**–**e** Heatmap showing the cumulative location of each 5–6 dpf control MO, *cep41* MO and human *CEP41* P206A and A107G mRNA injected target zebrafish larvae during 1 h experiment. **f** Quantified data presenting the percentage of time spent in each zone during 1 h experiment. **g** The statistical analysis of the time target larvae spent in each zone. The *cep41* MO and P206A larvae spend decreased amount of time in zone 1 adjacent to the group. The data are shown as the mean ± SD; ^*^p *<* 0.05 (Student’s *t*-test). All data are representative of at least five independent experiments

Next, we evaluated the effects of ASD variants identified in our case-control study on behaviors of 5–6 dpf zebrafish larvae. We found that control MOs-injected zebrafish exhibit preference for the zone 1 closest to a group (Fig. 3b). For zebrafish with *cep41* knockdown and p.P206A expression, the zone 1 preference was absent, whereas the larvae expressing p.A107G exhibited zone 1 preference in the same way as the control larvae (Fig. 3c–e). The zebrafish expressing an additional six variants, which were identified in ASD cases, lacked group preference similar to *cep41* morphants (Supplementary Figure 5). Further analysis of swimming velocity analysis did not show significant differences between *cep41*-depleted zebrafish larva or adults and controls (Supplementary Figure 6). Thus, this made it less likely that group preference deficits resulting from cep41 dysfunction are due to dampened swimming mechanics. Finally, the expression of WT *CEP41* or the p.A107G variant restored group preference behaviors in the *cep41*-silenced zebrafish (Supplementary Figure 7b and d), whereas expression of other variants, including p.P206A, identified in ASD cases failed to do so (Supplementary Figure 7c and e-j). Altogether, these data suggest that CEP41 is important in regulation of social interactions and the variants identified in ASD cases affect the behavioral traits.

## Discussion

Our study has shown that in addition to sporadic autism, NGS-based methods can be successfully applied to the identification of genes involved in familial autism. Our family-based exome sequencing coupled with stringent filtering for private protein altering variants (gene disrupting and missense) has resulted in a limited number of candidate genes. Using a systems biology approach and enrichment analysis, we have shown that candidate genes we identified are enriched for biological processes related to cell projection organization (GO:0030030) and neuron development (GO:0048666). The same biological processes were identified in enrichment analysis of SFARI ASD genes, validating that our candidate gene identification approach was effective. Cell projection organization and neuronal development have been identified as enriched in ASD before. Buxbaum et al. used a system-level analysis to evaluate 112 high-risk ASD genes^43^. A high-risk status was assigned to the genes as they are causally implicated in intellectual disability (ID) and the expressed phenotypes include ASD and autistic behaviors^44,45^. Our results expand on findings by Buxbaum et al.^43^, as the candidate genes we identified in enrichment analysis have not been associated with ASD before. In addition, genes in the reported analysis^43^ are all implicated in ID as well. In our study, subjects were ascertained for an ASD phenotype and on average the cognition was above ID cutoff, indicating that the genes we have identified might result in a more narrowly defined ASD phenotype. Furthermore, our enrichment analysis points to genes that are involved in MT cytoskeleton, cell junction and surface and kinase binding, further expanding the understanding of the biological basis of autism.

Our stringent filtering criteria that resulted in a small number of candidate genes in each family allowed us to take a candidate gene approach a step further. Based on Mendelian principles for gene discovery, and the hypothesis that in each family a single gene could be responsible for or be a major risk factor for ASD, we searched the literature to select 13 genes for further targeted sequencing and gene-based analysis in large case-control samples. In this way, we identified significant association of rare missense variants in *CEP41* with ASD. A prior resequencing study of candidate genes in ASD families positive for 7q32 linkage signals found rare missense and splice *CEP41* variants enriched in ASD cases as compared to controls^46^. In our study, we found statistically significant case-control association of rare *CEP41* variants with ASD, which was supported by family-based TDT association and significant preferential transmission of *CEP41* ASD variants to siblings. Furthermore, in a zebrafish model, we showed that these variants have functional effects on brain development and social behavior.

### How might *CEP41* contribute to autism?

The *CEP41* gene encodes a 41-kDa centrosomal protein that is conserved in vertebrates^38,47,48^. The gene is ubiquitously expressed in human organs including brain^49^. The centrosome is an organelle made of two centrioles that are formed by nine MT triplets embedded in a dense pericentriolar matrix (PCM)^50^. The centrosome serves as the main MT-organizing center of the cell and is a main regulator of cell cycle progression and cell division. In many cell types, following cell cycle division exit, the centrosome converts into a basal body and primary cilium, thus extending the centrosomal role to cell motility and polarity^51^. Centrosomes have been implicated in multiple processes during brain development including neurogenesis and neuronal migration^52^. Proteins that are part of the PCM and interact with MTs have been implicated in a broad spectrum of neurodevelopmental microcephalies (disorders with reduced brain size) and malformations of cortical development such as lissencephaly^53–56^.Therefore, the localization of *CEP41* in the MT-based cellular organelles including centrosomes implicates that MT could play a role in the pathogenesis of ASD. Given that MT dynamics is important in the migration of neural crest cells^57^, CEP41 may regulate the dynamic process of MT assembly/disassembly in the centrosome or the axon of neural crest cells during their migration.

In addition to centrosomes, *CEP41* involvement in ASD could be mediated through the protein’s role in cilia. The dysfunction of cilia is associated with a wide range of human diseases that are known as ciliopathies^58^. Cilia have a function in signal transduction, are ubiquitously expressed, and ciliary dysfunction encompasses most human organ systems with brain, sensory organs, kidneys, skeletal and endocrine/metabolic systems most frequently affected. The phenotypes resulting from ciliary dysfunction have been described as Joubert syndrome (JBTS MIM #213300), Meckel syndrome (MKS MIM #249000) and Bardet–Biedel syndrome (BBS MIM #209900). To date, more than 180 genes have been implicated in 35 established ciliopathies, with numbers continuing to grow. Homozygous *CEP41* splice-site mutations have been associated with JBTS, a neurodevelopmental ciliopathy characterized by hypotonia, developmental delays, distinctive MRI cerebellar and brain stem malformation, and a range of physical manifestations that can include ocular, renal, hepatic and endocrine abnormalities^38^. Furthermore, autism has been described as a part of the JBTS phenotype^59^ and genes associated with ciliary dysfunction have been reported in ASD as well (for example: *AHI1, CEP290, RPGRIP1L, GUCY2D, RPE65*)^44^.

Our study for the first time reported that *CEP41* is involved in axon development and CNC migration. We demonstrated that zebrafish carrying *CEP41* variants identified in ASD cases as well as *cep41* mutant/morphants have altered social behavior as manifested by their performance on a test of preference for social interaction. We further showed that these behavioral changes are not likely to be due to swimming abnormalities as we did not observe changes in swimming velocity for *cep41-*depleted zebrafish. The putative mechanism for how axonal and CNC migration deficits might cause ASD is through disruption in brain connectivity. Indeed, brain connectivity disruption has been implicated in ASD through neuroimaging and EEG connectivity studies^60,61^.

### Limitations and future directions

Although we identified the association of *CEP41* with ASD, our study design did not provide evidence for association of a specific candidate gene with autism in 25 out of 26 families. This is not surprising as ASD is highly heterogeneous and any repository, even if very large, may not be sufficiently enriched for a specific genetic subtype. We identified a total of 145 VOIs in 139 genes in 20 of 26 families and we have shown that the identified genes are enriched for biological processes such as neuronal development and cell projection organization that are implicated in ASD. Given the need for large number of cases and controls, it was only feasible to sequence 13 candidate genes for association analysis. Our enrichment analysis further supports that some of the 126 genes we have not evaluated in case-control study might be associated with ASD as well and this could be tested in future studies.

The involvement of *CEP41*, first in the ciliopathy JBTS and now with our study in ASD, points toward gene dosage and variant-specific effect as factors that determine the phenotype. In JBTS, the implicated variants are homozygous splice site-disrupting variants. In ASD, we have found that implicated variants are heterozygous and missense. Instances of different types of variants and gene dosage levels leading to distinctive phenotypes have been described in ciliopathies. For example, gene disrupting nonsense mutations in *CC2D2A* gene cause Meckel–Gruber syndrome (MKS) with severe phenotype^62^, whereas missense mutations in the same gene cause JBTS where the phenotype is less severe^63^. In BBS ciliopathy, gene dosage is an important modulator of phenotype. Retinal disease is one of the cardinal features in recessive BBS with homozygous mutations. Although heterozygous carriers do not have overt visual disability, electroretinography reveals decreased sensitivity of photoreceptor response in all parents of children with BBS^64^, indicating that gene dosage quantitatively affects the phenotype. For *CEP41*, the heterozygous state with one WT allele and one allele with a missense mutation might not perturb the biological system to the extent that it would cause severe neurodevelopment consequences such as in JBTS where both copies of the gene have a protein-disrupting mutation, but it could still lead to lower level of perturbation that is phenotypically expressed as ASD. Furthermore, as JBTS ciliopathies, including the *CEP41* subtype, are characterized by distinctive cerebellar malformation signature (i.e. molar tooth sign), the individuals with ASD and *CEP41* heterozygous mutations should be investigated with neuroimaging to evaluate for structural CNS abnormalities.

Our enrichment analysis also implies that other genetic mechanisms, besides single gene causality that we evaluated by case-control study, might be contributory for autism. The ciliopathies clearly demonstrate the involvement of a digenic mechanism^58,65^. The proteins coded by genes which are implicated in ciliopathies form complexes of physically closely interacting proteins^66^. Heterozygous defects in two closely interacting genes can cause biological disruption in a similar way as compound heterozygosity in a single gene. Indeed, a digenic mechanism has been implicated in *CEP41* ciliopathies; heterozygous missense *CEP41* variants coupled with heterozygous *KIF7* or *CC2D2A* variants were reported in individuals with ciliopathy^38^. A focus on ciliopathy genes and biologically defined molecular targets narrows the number of genes to be evaluated, thus significantly reducing the required sample size and burden of multiple testing for digenic mechanisms. In the future, involvement of a digenic mechanism for ciliopathy genes in ASD could be tested using currently publically available resources. For example, ASD genetics resources such as MSSNG (https://www.mss.ng/) and the Simons Simplex Collection (https://www.sfari.org/resource/simons-simplex-collection/) have a large number of exome and whole-genome sequenced autism cases and controls. For the approximately 180 genes associated with ciliopathies, one could evaluate whether digenic VOIs are seen more frequently in cases as compared to controls; and in such a way explore statistical evidence for a digenic mechanism in ASD.

## Supplementary information

Supplementary Information

Supplementary Table 5

Supplementary Table 6
