# Supplementary material for: Family-based exome sequencing and case-control analysis implicate CEP41 as an ASD gene
Source: Transl Psychiatry. 2019 Jan 15;9:4. doi: 10.1038/s41398-018-0343-z (PMC6341097; doi:10.1038/s41398-018-0343-z)

**Supplementary Information**

Pedigrees of the exome sequenced families

Family Id 152-HSC0079


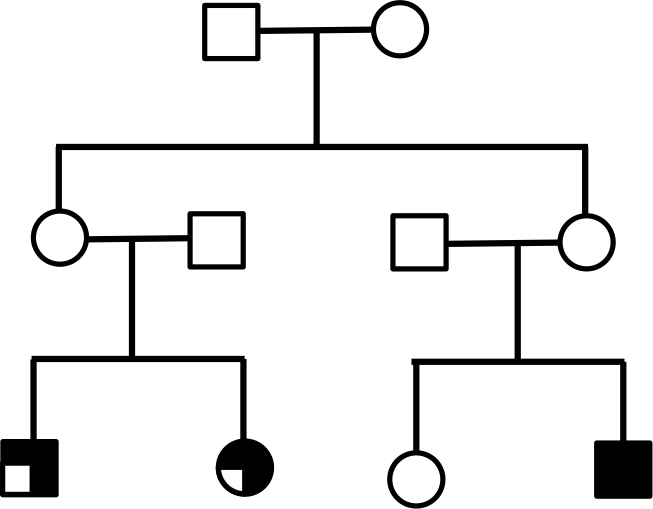


152-HSC0079-03 152-HSC0079-04 152-HSC0079-08

Family Id 156-3925


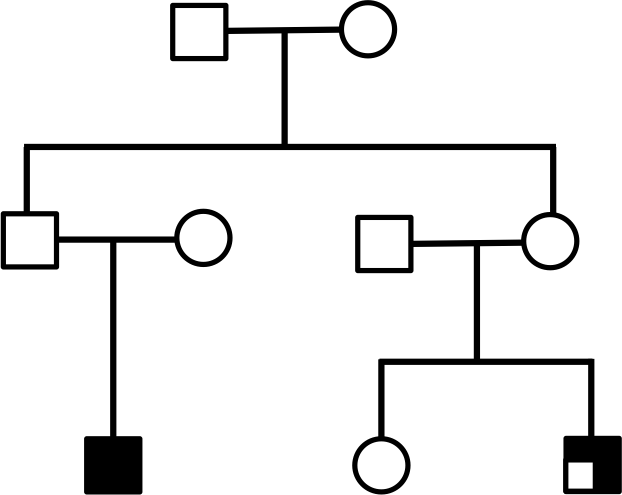


156-3925-351 156-3925-201

Family Id 60-1015


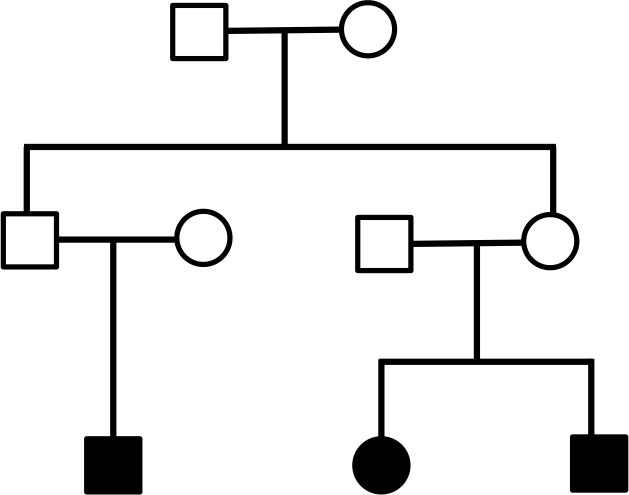


60-1015-002 60-1015-023

Family Id 60-1049


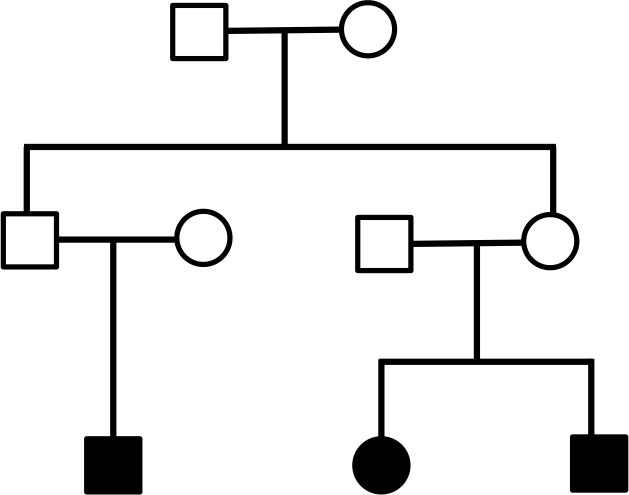


60-1049-003 60-1049-023 60-1049-024

Family Id 60-1057


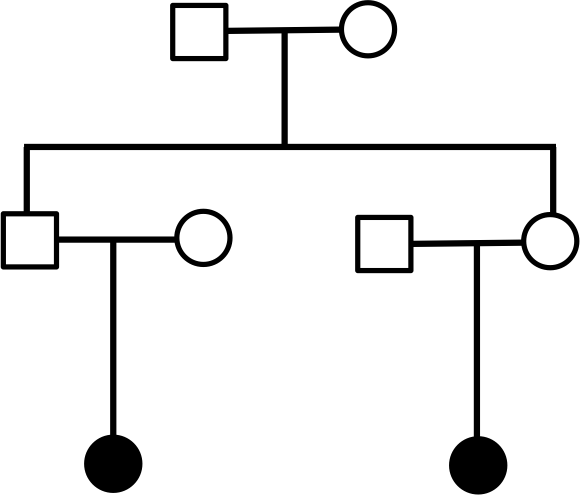


60-1057-004 60-1057-008

Family Id 60-2036


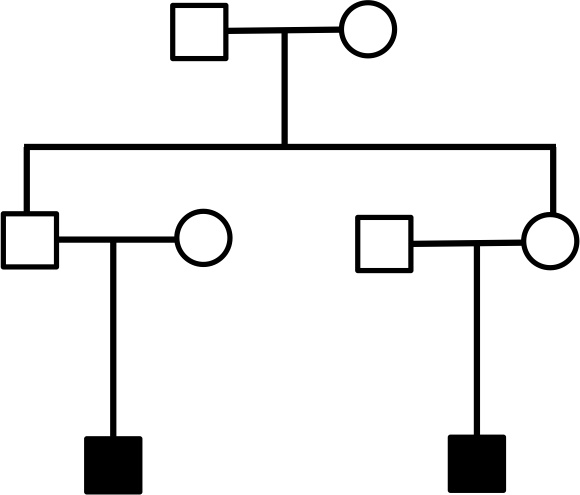


60-2036-008 60-2036-003

Family Id 60-4031


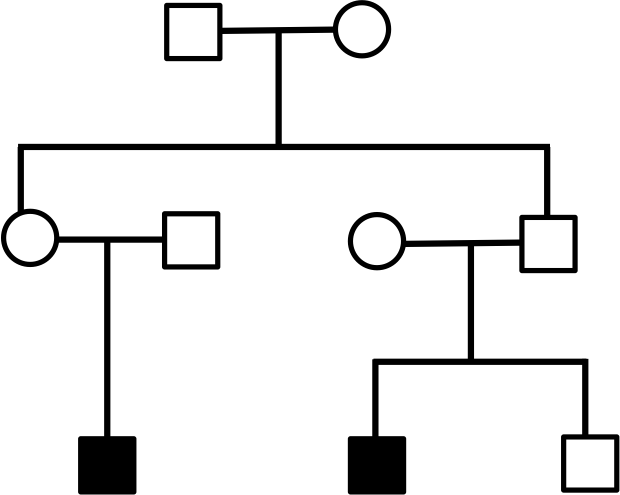


60-4031-001 60-4031-006

Family Id 61-2607


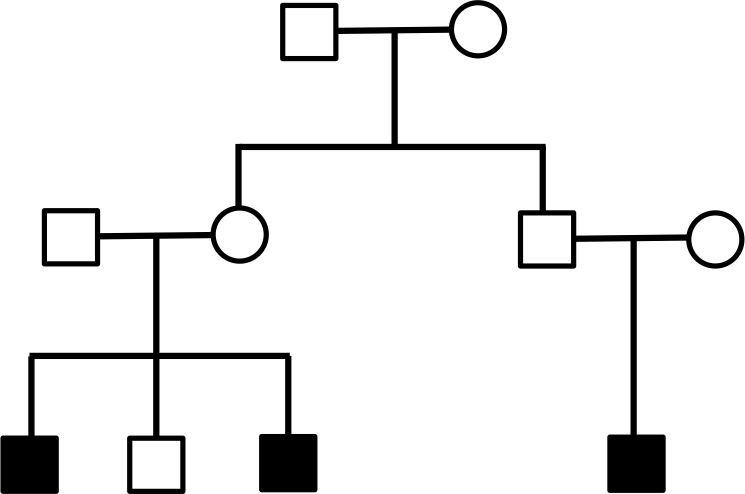


61-I-2607-005 61-I-2607-009

Family Id 63-0007


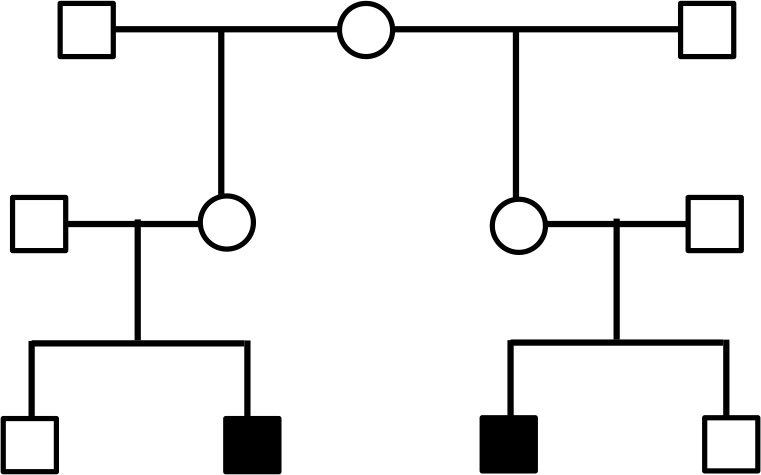


63-3183 63-2392

Family Id 72-0614


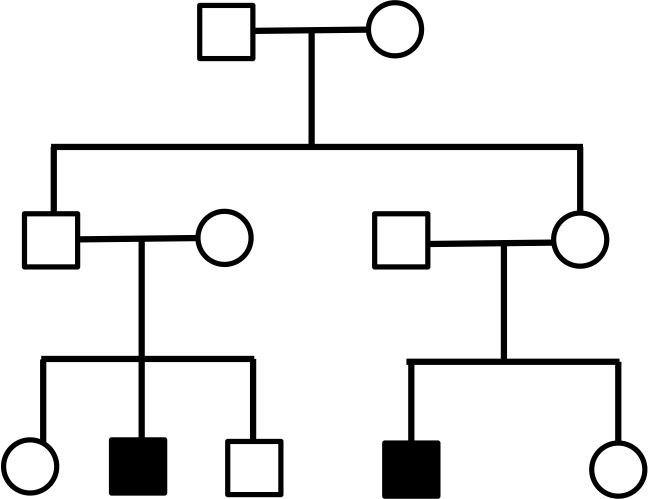


72-0614-04 72-0614-311

Family Id 72-0745


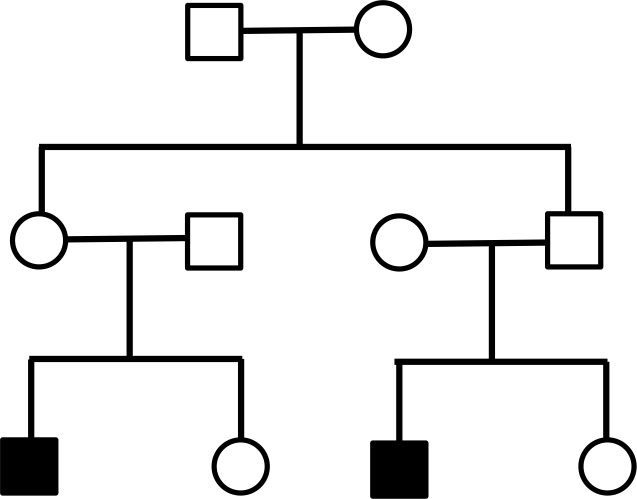


72-0745-03 72-0745-301

Family Id 72-1047


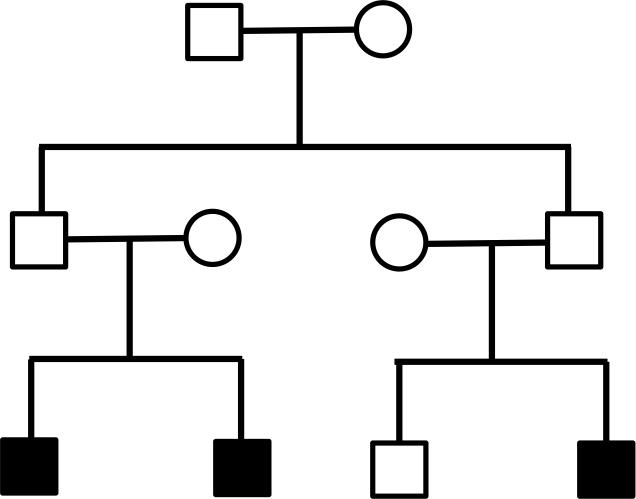


72-1047-301 72-1047-302 72-1047-312

Family Id 72-1245


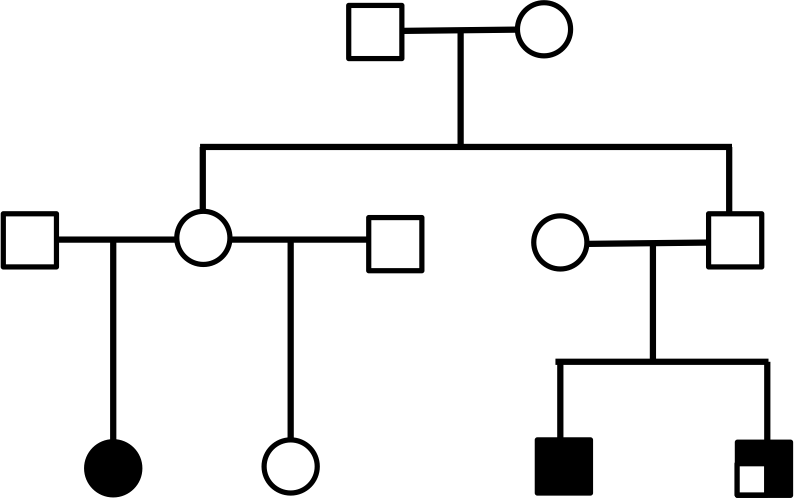


72-1245A-301 72-1245B-311 72-1245B-312

Family Id 72-1397


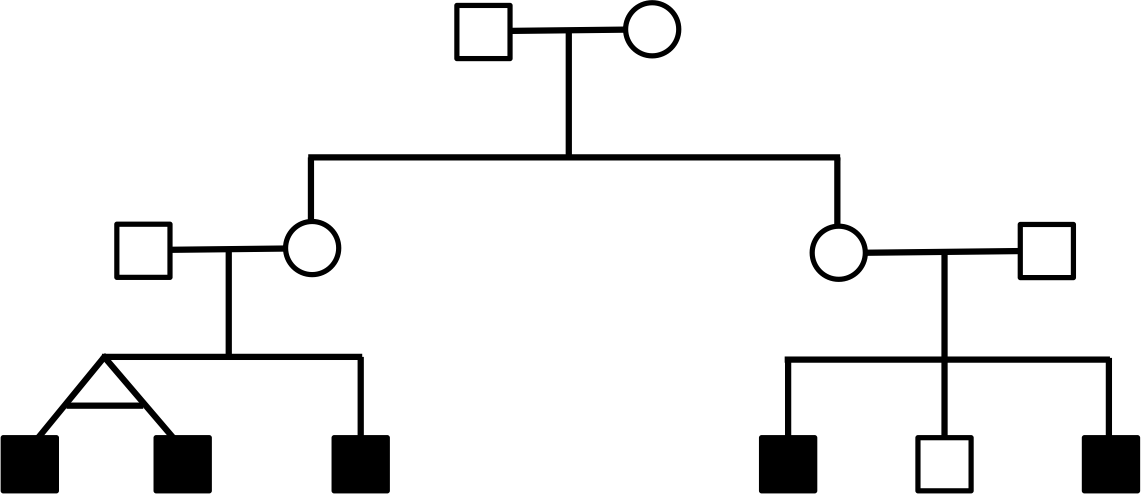


72-1397-302 72-1397-313

Family Id 72-1921


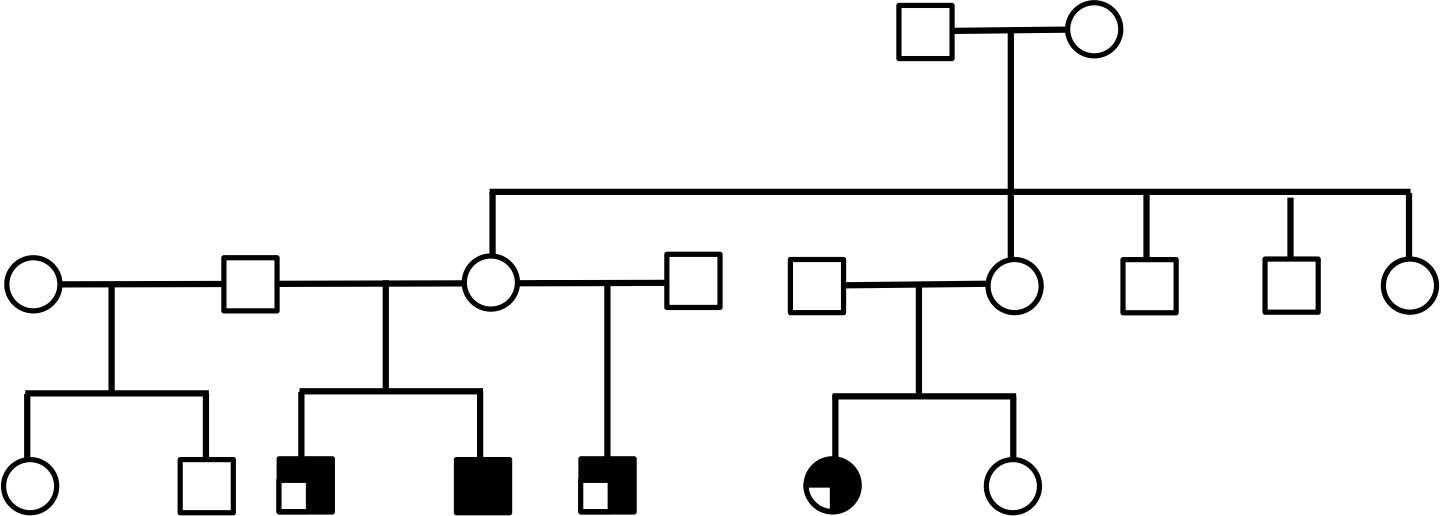


72-1921-305 72-1921-311

Family Id 74-0081


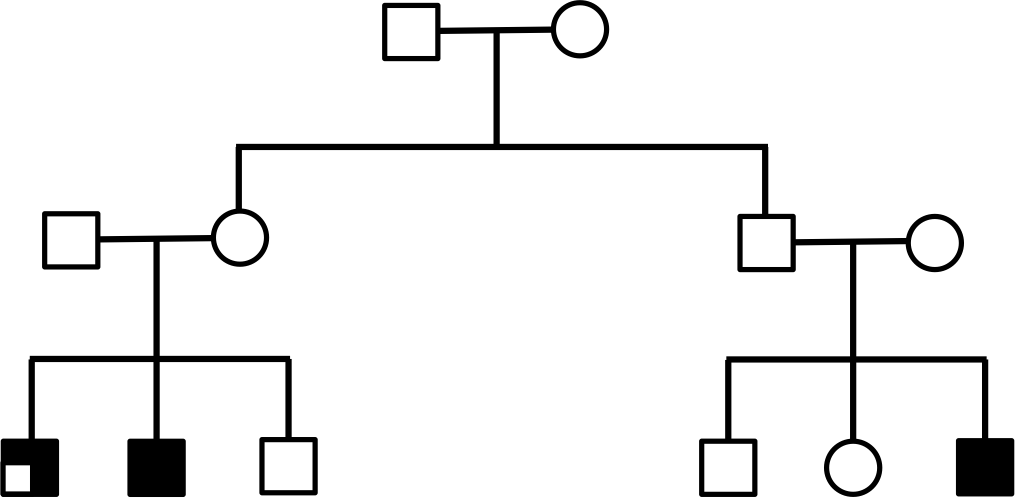


74-0081-03 74-0081-04 74-0081-303

Family Id 74-0180


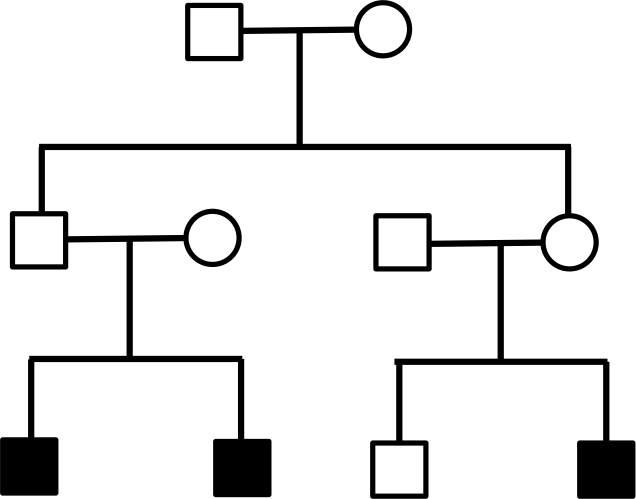


74-0180-03 74-0180-04 74-0180-10

Family Id 74-0264


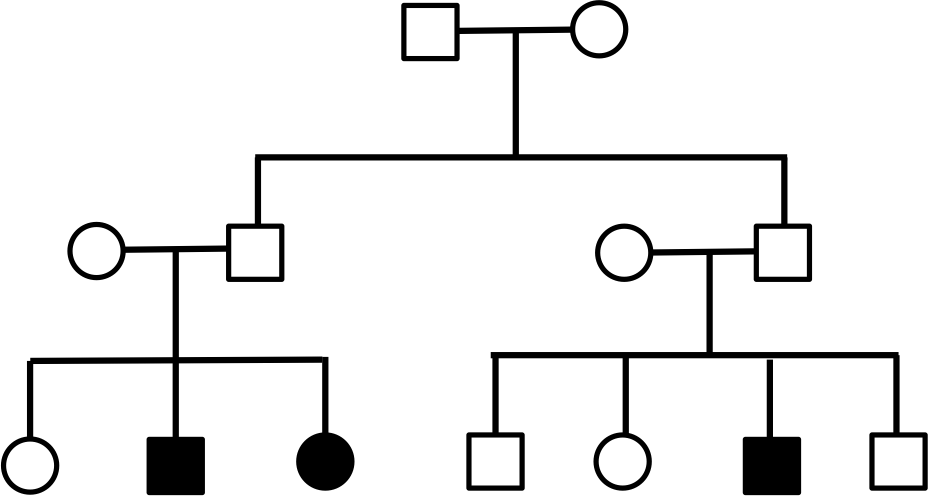


74-0264-04 74-0264-05 74-0264-12

Family Id 74-0358


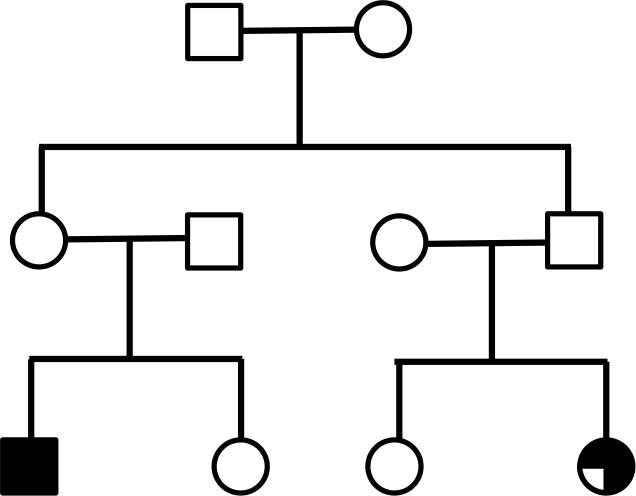


74-0358-10 74-0358-13

Family Id 74-0450


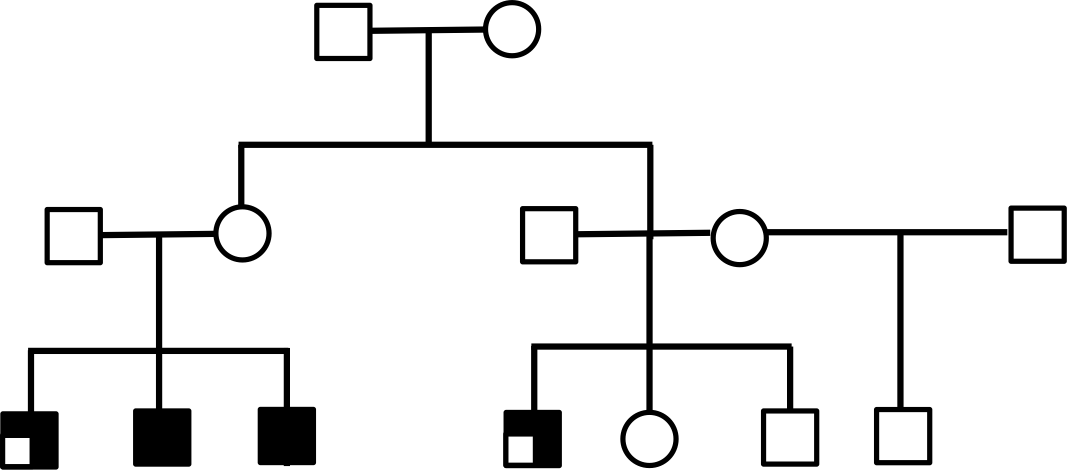


74-0450-04 74-0450-10

Family Id 74-0455


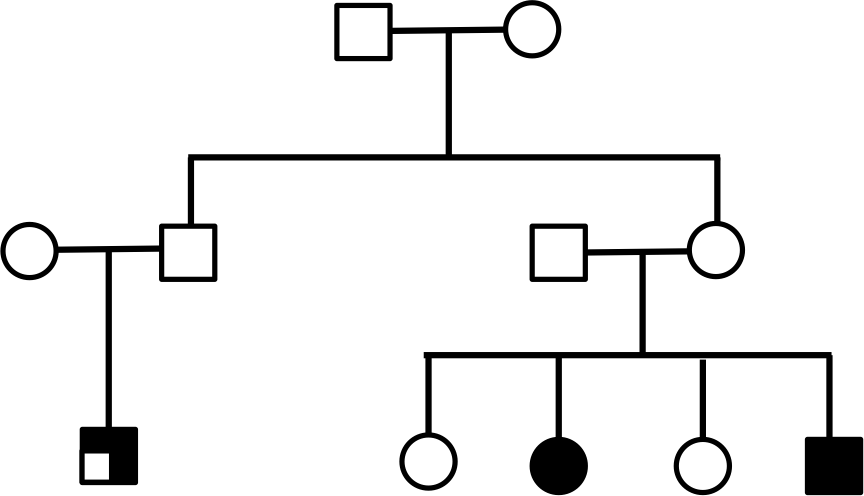


74-0455-10 74-0455-12 74-0455-14

Family Id 74-0481


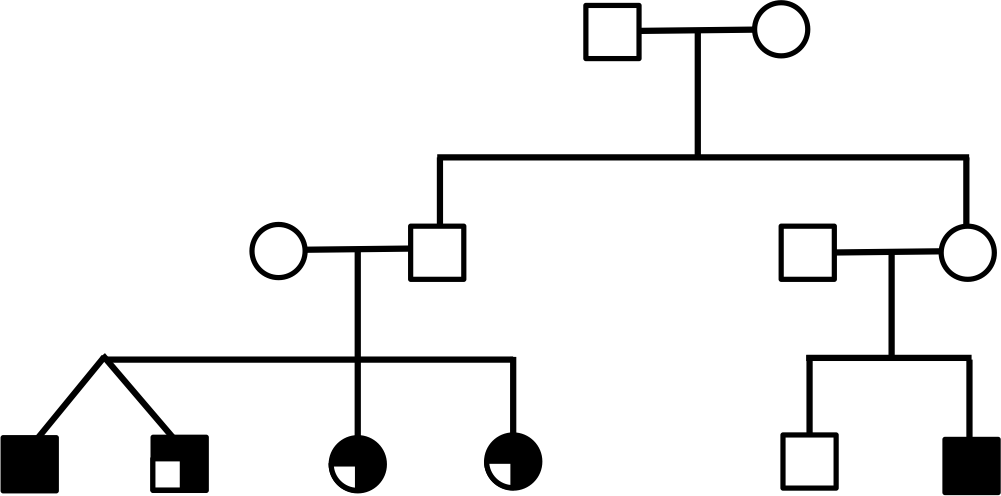


74-0481-03 74-0481-312

Family Id 74-0672


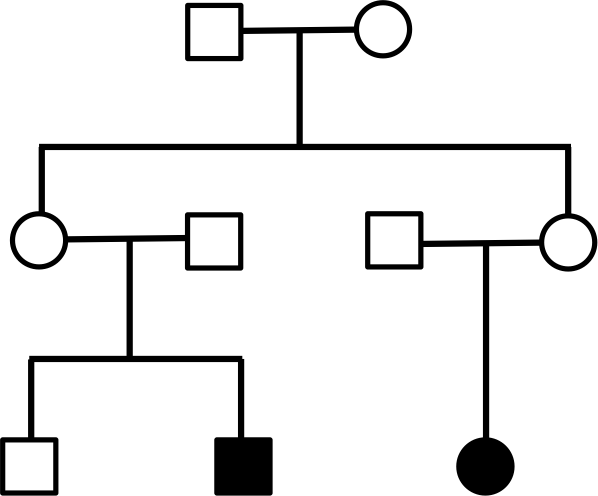


74-0672-08 74-0672-09

Family Id 74-0707


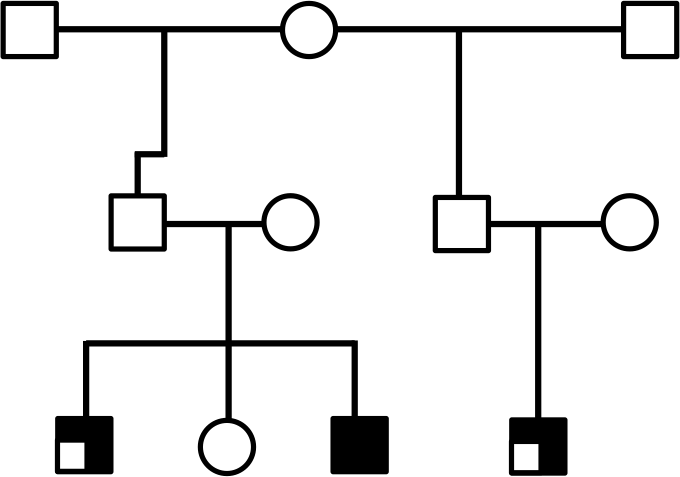


74-0707-03 74-0707-05 74-0707-10

Family Id 74-0752


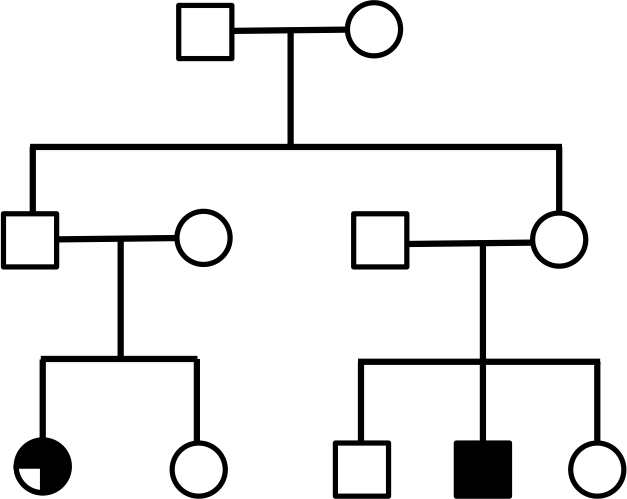


74-0752-07 74-0752-10

Family Id 74-0765


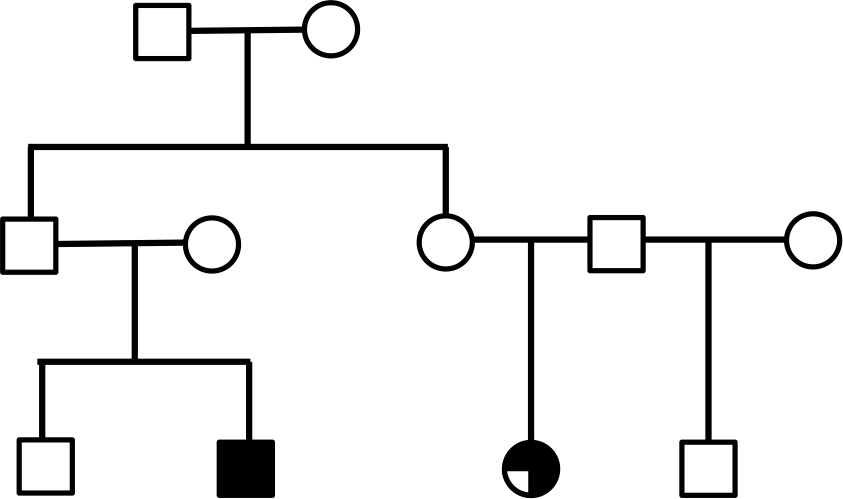


74-0765-08 74-0765-09


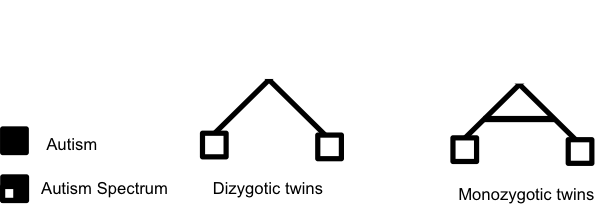


Supplementary Methods

**Exome Sequencing and Variant Identification**: Quality of the genomic DNA was checked prior to exome capture. For exome capture ~3.5 ug of high quality genomic DNA was fragmented, followed by end repair, A-tailing, sequencing adapter ligation, and PCR amplification. Exome capture was performed using the Nimblegen SeqCap EZ Human Exome Library v2.0 (Roche, Basel, Switzerland) following manufacturer's recommended protocol. Paired-end 50bp sequencing was performed on an Illumina HiSeq2000 sequencing platform in the University of Washington Genome Sciences Center for Mendelian Genomics. Base calling was done by CASAVA; subsequently sequenced reads were aligned to human reference genome GRCh37 (hg19) using Burrows-Wheeler Aligner (BWA v0.6.2) and BAM files were generated using SAMtools (v0.1.19). PCR duplicates were identified using Picard (v1.96) and removed. For calling single nucleotide variants (SNVs), Genome Analyzer Toolkit^4^ (GATK v3.2) was utilized. SNVs identified were further filtered by GATK VariantFiltration (low quality scores (≤50), allele balance (≥0.75), long homopolymer runs (>3), Filtered Read Depth 8, Quality by Depth 5.

**Targeted Capture Sequencing and Variant Identification**:

471 MIPs were designed with the MIP generator pipeline. The targeted sequence for each MIP was 112 bp long with 20 bp extension and ligation arms. Each MIP included a 5 bp long sequence tag as an identification barcode to separate up to 1024 unique reads from PCR duplicates. All 471 MIP probes were pooled and 5′ phosphorylated with T4 PNK (NEB). The phosphorylated MIP pool was combined with 100 ng of genomic DNA from each sample. The barcoded libraries were pooled, purified with AMPure XP (Agencourt) and sequenced using a paired end 101 bp multiplex protocol on HiSeq 2000 (illumina) at the Johns Hopkins Genetic Resources Core Facility (GRCF). Base calling on the sequenced reads was performed using CASAVA. PCR duplicates were filtered for each captured region for each sample and sequences were trimmed to remove the barcodes and MIP sequencing primers. The samples with >95% of targets covered with depth >8X were retained for downstream analysis. Sequence reads were aligned to NCBI human reference genome GRCh37 (hg19) using Burrows-Wheeler Aligner (BWA v0.7.10). Using Samtools^3^ (v0.1.19) reads were filtered and BAM files generated. We filtered out those reads which were not properly paired, were not mapping to expected locations and/ or were not having the expected mapping size. Sorting and indexing of the BAM files were performed using Picard (v1.118). Genome analysis toolkit (GATK) (v3.2) was used for local realignment and base call recalibration. GATK UnifiedGenotyper was used for calling the single nucleotide variants (SNVs). The identified SNVs were further filtered using GATK VariantFiltration. We removed individual genotypes if quality (GQ) was under 30, filtered depth (DP) was under 8 and allele balance (AB) was greater than 0.8. After such filtering, if more than 10% of individuals had a missing genotype, we excluded the entire site from analysis.

**Exome and Targeted Sequencing Variant Annotation**: We constructed a gene list that includes genes that are reported to frequently harbor false positive variants and also genes found to be false positive in our in-house database. For exome sequencing annotation, if the gene was reported to be in that list we excluded that gene from further consideration. To annotate variants based on frequency we used dbSNP132, 1000 Genomes (1KG) (2012April) and Exome Sequencing Project (ESP) Caucasian data sets. We defined variants identified in targeted sequencing analysis but with frequency < 0.01 in 1KG Caucasian samples as rare and variants identified in exome sequencing analysis but not present in any of the data sets as private. For statistical analysis we considered only protein altering variants (stop, frameshift, splice, and nonsynonymous).

Supplementary Tables

**Supplementary Table 1: Summary of exome sequencing for each sample.**

| Family Id | Subject Id | Total Reads | Target >8x | Target >20x | Mean Depth | SNP number |
| --- | --- | --- | --- | --- | --- | --- |
| 152-HSC0079 | 152-HSC0079-3 | 97233381 | 0.93451057 | 0.82393521 | 56.42798481 | 23228 |
|  | 152-HSC0079-4 | 85818954 | 0.936522797 | 0.828554169 | 51.33225965 | 23316 |
|  | 152-HSC0079-8 | 102940444 | 0.936280019 | 0.82941252 | 59.45840497 | 23738 |
| 156-3925 | 156-3925-201 | 98907300 | 0.929927484 | 0.813148439 | 56.61795212 | 23226 |
|  | 156-3925-351 | 121108873 | 0.931815627 | 0.824027558 | 64.32185993 | 22803 |
| 60-1015 | 60-1015-002 | 96452093 | 0.932569354 | 0.828510948 | 57.52308215 | 23100 |
|  | 60-1015-023 | 102368202 | 0.937523004 | 0.835698998 | 58.9070114 | 23020 |
| 60-1049 | 60-1049-003 | 99487672 | 0.934605613 | 0.826336507 | 57.43449025 | 23221 |
|  | 60-1049-023 | 108519176 | 0.932746484 | 0.829685645 | 61.6409292 | 25180 |
|  | 60-1049-024 | 99104191 | 0.933618198 | 0.823085977 | 57.26761197 | 24902 |
| 60-1057 | 60-1057-004 | 122395005 | 0.939485177 | 0.843029422 | 68.20558275 | 23838 |
|  | 60-1057-008 | 126037825 | 0.931872946 | 0.827010352 | 70.75186427 | 23698 |
| 60-2036 | 60-2036-003 | 108033061 | 0.938947392 | 0.841254585 | 61.9398457 | 23439 |
|  | 60-2036-008 | 102079501 | 0.941657904 | 0.843493204 | 58.82496201 | 23251 |
| 60-4031 | 60-4031-001 | 96299612 | 0.936319048 | 0.830321004 | 56.56857569 | 23122 |
|  | 60-4031-006 | 119804832 | 0.940814251 | 0.8475501 | 68.27096614 | 23268 |
| 61-2607 | 61-I-2607-005 | 102787486 | 0.93115324 | 0.82038148 | 58.9552551 | 23756 |
|  | 61-I-2607-009 | 102908185 | 0.931152723 | 0.819279045 | 58.03831871 | 23603 |
| 63-007 | 63-2392 | 113561125 | 0.929930369 | 0.824218024 | 63.8623027 | 23780 |
|  | 63-3183 | 103290416 | 0.934946346 | 0.827116309 | 59.07721498 | 23039 |
| 72-0614 | 72-0614-04 | 94533424 | 0.937296556 | 0.829462572 | 55.12899352 | 23624 |
|  | 72-0614-311 | 101201093 | 0.940073261 | 0.839820181 | 58.54031971 | 23511 |
| 72-0745 | 72-0745-03 | 114921078 | 0.929074032 | 0.818616577 | 59.75383852 | 23478 |
|  | 72-0745-301 | 104346459 | 0.928523237 | 0.815302059 | 59.02779225 | 23284 |
| 72-1047 | 72-1047-301 | 102251628 | 0.940590579 | 0.835439237 | 58.04259351 | 23158 |
|  | 72-1047-302 | 100786145 | 0.932877317 | 0.822103978 | 57.58704576 | 23225 |
|  | 72-1047-312 | 97182361 | 0.934902009 | 0.823367186 | 56.35811436 | 23261 |
| 72-1245 | 72-1245A-301 | 98787890 | 0.934294302 | 0.824059457 | 56.9367094 | 23683 |
|  | 72-1245A-311 | 98510564 | 0.933261053 | 0.820379085 | 56.16903384 | 23471 |
|  | 72-1245A-312 | 93608188 | 0.939727847 | 0.829934683 | 54.4408883 | 23502 |
| 72-1397 | 72-1397-302 | 132211452 | 0.916421056 | 0.843661407 | 98.77864384 | 23173 |
|  | 72-1397-313 | 153658678 | 0.925046477 | 0.866434195 | 118.795054 | 23873 |
| 72-1921 | 72-1921-305 | 186897032 | 0.911479872 | 0.842594082 | 136.1135811 | 23067 |
|  | 72-1921-311 | 138436616 | 0.913839445 | 0.840304512 | 102.8347914 | 23210 |
| 74-0081 | 74-0081-03 | 100481385 | 0.935357626 | 0.825189055 | 56.85075931 | 23551 |
|  | 74-0081-04 | 95300530 | 0.935123122 | 0.824580504 | 55.35583695 | 23295 |
|  | 74-0081-303 | 91390081 | 0.932643739 | 0.813726153 | 52.7001382 | 23156 |
| 74-0180 | 74-0180-03 | 93516067 | 0.935309533 | 0.821626016 | 53.83587645 | 23439 |
|  | 74-0180-04 | 99190610 | 0.936046903 | 0.825260146 | 56.60591001 | 23436 |
|  | 74-0180-10 | 94239586 | 0.934332923 | 0.82190434 | 54.28829741 | 23323 |
| 74-0264 | 74-0264-04 | 99996239 | 0.933727857 | 0.823998871 | 57.85633814 | 23193 |
|  | 74-0264-05 | 115672565 | 0.930876794 | 0.823772451 | 65.24209783 | 23525 |
|  | 74-0264-12 | 98950183 | 0.931593697 | 0.819115741 | 57.04625559 | 23526 |
| 74-0358 | 74-0358-10 | 106174894 | 0.933829649 | 0.825574179 | 61.10825182 | 24386 |
|  | 74-0358-13 | 93961466 | 0.931133998 | 0.815870002 | 54.4095133 | 23110 |
| 74-0450 | 74-0450-04 | 143940652 | 0.916146869 | 0.846699452 | 108.745242 | 22990 |
|  | 74-0450-10 | 147258578 | 0.932266753 | 0.878229447 | 112.4193652 | 23561 |
| 74-0455 | 74-0455-10 | 101759918 | 0.936271854 | 0.828039246 | 58.29867832 | 23402 |
|  | 74-0455-12 | 97385158 | 0.937830232 | 0.833559504 | 55.95242829 | 23349 |
|  | 74-0455-14 | 99117018 | 0.934674881 | 0.824621194 | 56.73442644 | 23452 |
| 74-0481 | 74-0481-03 | 93767736 | 0.919424181 | 0.835978384 | 71.58274968 | 23059 |
|  | 74-0481-312 | 111024456 | 0.890735994 | 0.78773966 | 82.22414375 | 22859 |
| 74-0672 | 74-0672-08 | 103328069 | 0.94107853 | 0.845523394 | 59.89211096 | 23197 |
|  | 74-0672-09 | 121422417 | 0.933115196 | 0.82902892 | 68.16452772 | 23687 |
| 74-0707 | 74-0707-03 | 102392914 | 0.935340397 | 0.823104621 | 57.84132129 | 23330 |
|  | 74-0707-05 | 90012059 | 0.934832523 | 0.819916119 | 52.79968172 | 23331 |
|  | 74-0707-10 | 91474084 | 0.93684328 | 0.824382716 | 52.56916396 | 22902 |
| 74-0752 | 74-0752-07 | 100996851 | 0.935451308 | 0.832681094 | 58.41540366 | 23927 |
|  | 74-0752-10 | 115858579 | 0.933770043 | 0.819341019 | 63.80279323 | 24176 |
| 74-0765 | 74-0765-08 | 105529546 | 0.930353515 | 0.818764013 | 59.88421052 | 23144 |
|  | 74-0765-09 | 107754803 | 0.926857268 | 0.81629372 | 60.30579228 | 23204 |

**Supplementary Table 2: Private variants shared by the affected cousins of each exome-sequenced family.**

**SIFT**(Sorting Intolerant From Tolerant) predicts impact of amino acid substitutions based on the degree of conservation in sequence alignments derived from closely related sequences. Scores <0.05 are considered deleterious. **PolyPhen-2**(Polymorphism Phenotyping v2) predicts impact of a variant on the structure and function of a human protein using eight sequence-based and three structure-based predictive features. Scores >0.95 are considered probably damaging. **GERP** (Genomic Evolutionary Rate Profiling) identifies functional constraint of a sequence variant by quantifying substitution deficits in multiple alignments. Substitution deficits represent a natural measure of constraint that reflects the strength of past purifying selection. Higher GERP scores are more deleterious. Genes selected for case control analysis are represented in bold front.

| Family | Position | Ref/Alt | Gene | | SNV Type | AAChange | | SIFT | | | Polyphen2 | | GERP | |  |  |
| --- | --- | --- | --- | --- | --- | --- | --- | --- | --- | --- | --- | --- | --- | --- | --- | --- |
| 152-HSC0079 | chr5:178553060 | C/T | ADAMTS2 | | Nonsynonymous | p.A897T | | 0.58 | | | 0.002 | | -1.61 | |  |  |
| 152-HSC0079 | chr11:118428568 | C/T | IFT46 | | Nonsynonymous | p.R28K | | 0.38 | | | 0 | | 3.89 | |  |  |
| 152-HSC0079 | chr11:34502431 | C/T | ELF5 | | Nonsynonymous | p.D92N | | 0.36 | | | 0.999 | | 5.7 | |  |  |
| 152-HSC0079 | chr19:1796779 | G/A | ATP8B3 | | Nonsynonymous | p.R515W | | 0.09 | | | 1 | | 2.52 | |  |  |
| 152-HSC0079 | chr2:160964212 | C/T | ITGB6 | | Nonsynonymous | p.R654Q | | 0.05 | | | 0.994 | | 5.79 | |  |  |
| 152-HSC0079 | chr13:99361005 | C/G | SLC15A1 | | Nonsynonymous | p.A362P | | 0.02 | | | 0.092 | | 5.32 | |  |  |
| 152-HSC0079 | chr13:70514228 | A/T | KLHL1 | | Nonsynonymous | p.F259I | | 0.02 | | | 1 | | -5.67 | |  |  |
| 152-HSC0079 | chr11:66252653 | G/C | DPP3 | | Nonsynonymous | p.V94L | | 0 | | | 0.022 | | 4.08 | |  |  |
| 152-HSC0079 | chr13:25487103 | T/A | CENPJ | | Nonsynonymous | p.M21L | | 0 | | | 0.647 | | 5.28 | |  |  |
| 152-HSC0079 | chr11:66082328 | T/G | CD248 | | Nonsynonymous | p.D724A | | 0 | | | 0.997 | | 3.48 | |  |  |
| 152-HSC0079 | chr5:167841447 | G/A | WWC1 | | Nonsynonymous | p.E346K | | 0 | | | 1 | | 5.68 | |  |  |
| 152-HSC0079 | chr10:135107082 | A/T | TUBGCP2 | | Nonsynonymous | p.Y140N | | 0 | | | 1 | | 4.26 | |  |  |
| 152-HSC0079 | chr11:34475453 | T/G | CAT | | Nonsynonymous | p.Y231D | | 0 | | | 1 | | 6.06 | |  |  |
| 156-3925 | chr13:52951937 | T/C | THSD1 | | Nonsynonymous | p.N670S | | 0.89 | | | 0 | | 1.44 | |  |  |
| 156-3925 | chr10:127460819 | C/T | MMP21 | | Stopgain | p.W316X | | 0.28 | | | . | | 4.22 | |  |  |
| 156-3925 | chr1:10479487 | G/A | PGD | | Nonsynonymous | p.R408Q | | 0.25 | | | 0.016 | | 4.96 | |  |  |
| 156-3925 | chr14:75248524 | C/A | YLPM1 | Nonsynonymous | | | p.P593Q | | 0.16 | 0.993 | | | 4.85 | | | |
| 156-3925 | **chr7:130039895** | **G/C** | **CEP41** | **Nonsynonymous** | | | **p.P320A** | | **0.14** | **0.598** | | | **5.64** | | | |
| 156-3925 | chr22:20761112 | T/A | ZNF74 | Nonsynonymous | | | p.Y526N | | 0.01 | 0.244 | | | 2.72 | | | |
| 156-3925 | chr13:41517148 | A/G | ELF1 | Nonsynonymous | | | p.L225S | | 0.01 | 1 | | | -4.47 | | | |
| 156-3925 | chr1:224491525 | G/A | NVL | Nonsynonymous | | | p.P71L | | 0 | 1 | | | 4.96 | | | |
| 60-1015 | chr4:187153307 | G/A | KLKB1 | Nonsynonymous | | | p.A29T | | 1 | 0.001 | | | 0.062 | | | |
| 60-1015 | chr20:45644877 | A/G | EYA2 | Nonsynonymous | | | p.Y119C | | 0.19 | 1 | | | -5.82 | | | |
| 60-1015 | chr15:45335545 | A/G | SORD | Nonsynonymous | | | p.K64R | | 0.15 | 0.001 | | | 3.61 | | | |
| 60-1015 | **chr1:16074127** | **A/G** | **TMEM82** | **Stoploss** | | | **p.X344W** | | **.** | **.** | | | **2.42** | | | |
| 60-1057 | chr3:48456740 | A/C | PLXNB1 | Nonsynonymous | | | p.C1271G | | 0.22 | 0.001 | | | -3.07 | | | |
| 60-1057 | chr12:52695854 | G/C | KRT86 | Nonsynonymous | | | p.G52R | | 0.03 | 0.99 | | | 5 | | | |
| 60-1057 | chr12:283924 | C/G | IQSEC3 | Nonsynonymous | | | p.Q1092E | | 0.02 | 0.956 | | | 3.67 | | | |
| 60-1057 | chr11:4566422 | T/C | OR52M1 | Nonsynonymous | | | p.M1T | | 0 | 0.791 | | | 2.76 | | | |
| 60-1057 | chr3:27297817 | G/A | NEK10 | Nonsynonymous | | | p.T687I | | . | . | | | 4.96 | | | |
| 60-2036 | **chr2:235950057** | **A/G** | **SH3BP4** | **Nonsynonymous** | | | **p.N215S** | | **0.61** | **0.011** | | | **5.58** | | | |
| 60-2036 | chr17:66364696 | G/A | ARSG | Nonsynonymous | | | p.G238R | | 0.12 | | | 0.059 | | -2.25 | |  |
| 60-2036 | chr17:56281589 | G/C | EPX | Nonsynonymous | | | p.W651C | | 0 | | | 1 | | 5.65 | |  |
| 60-2036 | chr12:105460408 | G/A | ALDH1L2 | | Nonsynonymous | p.R212C | | . | | | 0.032 | | 4.02 | |  |  |
| 60-4031 | chr14:24528890 | G/A | LRRC16B | | Nonsynonymous | p.R606Q | | 0.22 | | | 0.962 | | 5.04 | |  |  |
| 60-4031 | chr13:20413118 | C/G | ZMYM5 | | Nonsynonymous | p.W198C | | 0.08 | | | 0.89 | | 3.82 | |  |  |
| 60-4031 | chr17:36963226 | G/A | CWC25 | | Nonsynonymous | p.R232W | | 0.01 | | | 0.999 | | 2.96 | |  |  |
| 60-4031 | chr12:49433291 | C/G | KMT2D | | Nonsynonymous | p.S2719T | | 0 | | | 0.044 | | -2 | |  |  |
| 60-4031 | chr11:118981302 | T/A | C2CD2L | | Nonsynonymous | p.V161E | | 0 | | | 0.616 | | 4.84 | |  |  |
| 60-4031 | chr6:117116988 | G/C | GPRC6A | | Nonsynonymous | p.S449C | | 0 | | | 1 | | 4.99 | |  |  |
| 60-4031 | chr12:112036756 | T/G | ATXN2 | | Nonsynonymous | p.Q188P | | . | | | 0 | | -5.84 | |  |  |
| 72-0745 | chr7:33015967 | C/T | FKBP9 | | Nonsynonymous | p.H187Y | | 1 | | | 0.117 | | -4.73 | |  |  |
| 72-0745 | chr19:14001149 | G/A | C19orf57 | | Stopgain | p.Q174X | | 1 | | | . | | -0.805 | |  |  |
| 72-0745 | chr5:140308176 | G/C | PCDHAC1 | | Nonsynonymous | p.V567L | | 0.8 | | | 0.659 | | 5.95 | |  |  |
| 72-0745 | chr2:220039823 | A/G | CNPPD1 | | Nonsynonymous | p.C90R | | 0.57 | | | 0.93 | | 4.43 | |  |  |
| 72-0745 | chr4:152570812 | C/T | FAM160A1 | | Nonsynonymous | p.T540M | | 0.26 | | | 0.012 | | 1.72 | |  |  |
| 72-0745 | chr2:210560711 | T/A | MAP2 | | Nonsynonymous | p.C1273S | | 0.24 | | | 0.01 | | 4.4 | |  |  |
| 72-0745 | chr11:46408062 | C/T | CHRM4 | | Nonsynonymous | p.V16M | | 0.19 | | | 0.007 | | 3.84 | |  |  |
| 72-0745 | chr11:61665796 | T/C | RAB3IL1 | | Nonsynonymous | p.K342R | | 0.16 | | | 0.039 | | 3.56 | |  |  |
| 72-0745 | chr3:39171613 | C/A | TTC21A | | Nonsynonymous | p.L686M | | 0.05 | | | 1 | | 3.19 | |  |  |
| 72-0745 | chr7:89874755 | C/G | C7orf63 | | Nonsynonymous | p.A6G | | 0.04 | | | 0.419 | | 3.73 | |  |  |
| 72-0745 | chr17:4799506 | C/T | MINK1 | | Nonsynonymous | p.H1125Y | | 0.04 | | | 0.925 | | 4.71 | |  |  |
| 72-0745 | chr5:139745591 | G/A | SLC4A9 | | Nonsynonymous | p.R614H | | 0 | | | 0.027 | | 1.68 | |  |  |
| 72-0745 | chr5:132534876 | A/G | FSTL4 | | Nonsynonymous | p.F814L | | 0 | | | 0.122 | | 3.99 | |  |  |
| 72-0745 | chr7:82763882 | G/A | PCLO | | Nonsynonymous | p.P995L | | 0 | | | 0.649 | | 6.07 | |  |  |
| 72-0745 | chr10:88730270 | C/G | ADIRF | | Nonsynonymous | p.I54M | | 0 | | | 0.6 | | -0.349 | |  |  |
| 72-0745 | chr7:143088585 | G/A | EPHA1 | | Nonsynonymous | p.R966C | | 0 | | | 1 | | 5.24 | |  |  |
| 72-0745 | chr12:112036756 | T/G | ATXN2 | | Nonsynonymous | p.Q188P | | . | | | 0 | | -5.84 | |  |  |
| 72-1047 | chr5:71493249 | C/T | MAP1B | | Nonsynonymous | p.P1356L | | 0.23 | | | 0.005 | | 5.72 | |  |  |
| 72-1047 | **chr18:77208917** | **G/A** | **NFATC1** | | **Nonsynonymous** | **p.E36K** | | **0** | | | **1** | | **4.97** | |  |  |
| 72-1245 | **chr20:1286109** | **G/T** | **SNPH** | | **Nonsynonymous** | **p.G299V** | | **0.52** | | | **0.733** | | **0.214** | |  |  |
| 72-1245 | chr14:24724388 | G/A | TGM1 | | Nonsynonymous | p.R573W | | 0.02 | | | 0.999 | | 4.16 | |  |  |
| 72-1245 | chr14:24621007 | C/T | RNF31 | | Nonsynonymous | p.R646W | | 0 | | | 1 | | 3.93 | |  |  |
| 72-1397 | chr10:6527171 | G/T | PRKCQ | | Nonsynonymous | p.P196T | | 0.51 | | | 0.085 | | 4.26 | |  |  |
| 72-1397 | chr3:136665003 | A/G | NCK1 | | Nonsynonymous | p.I205V | | 0.28 | | | 0 | | 4.92 | |  |  |
| 72-1397 | chr11:64323737 | G/C | SLC22A11 | | Nonsynonymous | p.R89P | | 0.28 | | | 1 | | -4.04 | |  |  |
| 72-1397 | chr7:31917646 | C/G | PDE1C | | Nonsynonymous | p.M143I | | 0.21 | | | 0.974 | | 5.75 | |  |  |
| 72-1397 | chr5:52899293 | C/G | NDUFS4 | | Nonsynonymous | p.T37S | | 0.18 | | | 0.908 | | 4 | |  |  |
| 72-1397 | **chr6:24178621** | **A/T** | **DCDC2** | | **Nonsynonymous** | **p.N421K** | | **0.17** | | | **0.253** | | **0.05** | |  |  |
| 72-1397 | chr6:40400491 | C/T | LRFN2 | | Nonsynonymous | p.R121Q | | 0.16 | | | 0.801 | | 5.76 | |  |  |
| 72-1397 | chr6:35210840 | G/A | SCUBE3 | | Nonsynonymous | p.R579Q | | 0.06 | | | 0.143 | | 5.08 | |  |  |
| 72-1397 | chr12:977433 | G/C | WNK1 | | Nonsynonymous | p.Q847H | | 0.03 | | | 0.891 | | 2.85 | |  |  |
| 72-1397 | chr9:99522256 | C/A | ZNF510 | | Stopgain | p.E286X | | 0.02 | | | . | | 0.943 | |  |  |
| 72-1397 | chr17:27963290 | G/T | SSH2 | | Nonsynonymous | p.S626Y | | 0.01 | | | 0.999 | | 6.17 | |  |  |
| 72-1397 | chr2:99863200 | T/C | LYG2 | | Nonsynonymous | p.I43V | | . | | | 0.004 | | 1.6 | |  |  |
| 72-1921 | chr21:47666565 | G/A | MCM3AP | | Nonsynonymous | p.A1509V | | 0.76 | | | 0.017 | | 2.78 | |  |  |
| 72-1921 | **chr20:34065858** | **G/A** | **CEP250** | | **Nonsynonymous** | **p.A676T** | | **0.32** | | | **0.002** | | **2.37** | |  |  |
| 72-1921 | chr3:50357678 | G/T | HYAL2 | | Nonsynonymous | p.D81E | | 0.24 | | | 0.017 | | -2.5 | |  |  |
| 72-1921 | chr1:6530645 | T/C | PLEKHG5 | | Nonsynonymous | p.E534G | | 0.17 | | | 1 | | 4.75 | |  |  |
| 72-1921 | chr1:32160798 | G/A | COL16A1 | | Nonsynonymous | p.R324W | | 0.04 | | | 0.968 | | 2.16 | |  |  |
| 72-1921 | chr1:32829817 | T/C | TSSK3 | | Nonsynonymous | p.I256T | | 0 | | | 0.205 | | 5.5 | |  |  |
| 72-1921 | chr16:68011584 | T/C | DPEP3 | | Nonsynonymous | p.N327S | | 0 | | | 1 | | -4.71 | |  |  |
| 74-0081 | chr7:4830311 | C/T | AP5Z1 | | nonsynonymous | p.A649V | | 0.6 | | | 0.911 | | 5.3 | |  |  |
| 74-0081 | **chr16:15141730** | **C/A** | **NTAN1** | | **nonsynonymous** | **p.V78L** | | **0.32** | | | **0.011** | | **4.44** | |  |  |
| 74-0081 | chr6:3140676 | G/A | BPHL | | nonsynonymous | p.G241S | | 0.03 | | | 1 | | 5.56 | |  |  |
| 74-0081 | chr7:117267592 | G/A | CFTR | | nonsynonymous | p.R1162Q | | . | | | . | | 5.77 | |  |  |
| 74-0180 | chr6:41248867 | T/C | TREM1 | | nonsynonymous | p.N144S | | 0.78 | | | 0 | | -6.99 | |  |  |
| 74-0180 | chr1:158986366 | G/A | IFI16 | | nonsynonymous | p.S142N | | 0.43 | | | 0.006 | | -4.85 | |  |  |
| 74-0180 | **chr2:231865140** | **G/T** | **SPATA3** | | **nonsynonymous** | **p.A121S** | | **0.12** | | | **0.998** | | **3.25** | |  |  |
| 74-0180 | chr1:160147313 | G/T | ATP1A4 | | nonsynonymous | p.M1I | | 0 | | | 0.862 | | -4.29 | |  |  |
| 74-0358 | chr13:29286840 | C/T | SLC46A3 | | nonsynonymous | p.S346N | | 0.35 | | | 0.005 | | -0.805 | |  |  |
| 74-0358 | chr2:234622043 | G/T | UGT1A5 | | nonsynonymous | p.A136S | | 0.13 | | | 0.001 | | -8.97 | |  |  |
| 74-0358 | chr13:28001292 | C/T | GTF3A | | nonsynonymous | p.R89C | | 0.06 | | | 1 | | 5.05 | |  |  |
| 74-0358 | chrX:114424585 | T/G | RBMXL3 | | nonsynonymous | p.L194W | | 0 | | | 0.996 | | -0.369 | |  |  |
| 74-0358 | chr22:19958847 | G/C | ARVCF | | nonsynonymous | p.S931R | | . | | | 0.016 | | 3.95 | |  |  |
| 74-0358 | chr22:19958848 | C/A | ARVCF | | nonsynonymous | p.S931I | | . | | | 0.008 | | 3.95 | |  |  |
| 74-0450 | chr4:146824346 | G/A | ZNF827 | | nonsynonymous | p.A22V | | 0.28 | | | 0.006 | | 3.63 | |  |  |
| 74-0450 | **chr3:38797370** | **T/C** | **SCN10A** | | **nonsynonymous** | **p.E457G** | | **0.28** | | | **0.732** | | **4.71** | |  |  |
| 74-0450 | chr11:64323737 | G/C | SLC22A11 | | nonsynonymous | p.R89P | | 0.28 | | | 1 | | -4.04 | |  |  |
| 74-0450 | chr1:3391344 | A/G | ARHGEF16 | | nonsynonymous | p.K460E | | 0.18 | | | 0.571 | | 3.51 | |  |  |
| 74-0450 | chr22:19165466 | C/G | SLC25A1 | | nonsynonymous | p.K104N | | 0 | | | 0.999 | | 3.25 | |  |  |
| 74-0450 | chr12:2994578 | C/G | RHNO1 | | nonsynonymous | p.L16V | | 0 | | | 1 | | 4.44 | |  |  |
| 74-0450 | chr1:79357261 | T/C | ELTD1 | | nonsynonymous | p.N653S | | . | | | 1 | | 5.59 | |  |  |
| 74-0481 | chr17:17050657 | G/A | MPRIP | | nonsynonymous | p.A411T | | 1 | | | 0.106 | | -5.4 | |  |  |
| 74-0481 | chr17:16335605 | G/A | TRPV2 | | Stopgain | p.W660X | | 1 | | | . | | 4.85 | |  |  |
| 74-0481 | chr5:176524669 | C/T | FGFR4 | | Stopgain | p.Q761X | | 0.7 | | | . | | 3.39 | |  |  |
| 74-0481 | chr1:223284125 | T/C | TLR5 | | nonsynonymous | p.N750S | | 0.57 | | | 0 | | 2.9 | |  |  |
| 74-0481 | chr20:47865387 | C/T | ZNFX1 | | nonsynonymous | p.E1392K | | 0.26 | | | 0.177 | | 5.18 | |  |  |
| 74-0481 | chr8:70650428 | C/T | SLCO5A1 | | nonsynonymous | p.A424T | | 0.24 | | | 1 | | 5.81 | |  |  |
| 74-0481 | chr1:6530645 | T/C | PLEKHG5 | | nonsynonymous | p.E534G | | 0.17 | | | 1 | | 4.75 | |  |  |
| 74-0481 | chr7:100284283 | G/C | GIGYF1 | | nonsynonymous | p.S228C | | 0.04 | | | 0 | | 4.05 | |  |  |
| 74-0481 | chr9:35547963 | C/T | RUSC2 | | nonsynonymous | p.T482M | | 0.02 | | | 0.987 | | 4.47 | |  |  |
| 74-0481 | chr6:153296228 | C/A | FBXO5 | | nonsynonymous | p.R165L | | 0.01 | | | 1 | | -5.92 | |  |  |
| 74-0481 | chr21:31864073 | C/T | KRTAP19-3 | | nonsynonymous | p.R68H | | 0 | | | 0.002 | | -4.45 | |  |  |
| 74-0481 | chr17:1540104 | T/C | SCARF1 | | nonsynonymous | p.E511G | | 0 | | | 1 | | 5.15 | |  |  |
| 74-0481 | chr13:24468278 | C/A | C1QTNF9B | | Stopgain | p.G77X | | . | | | . | | 3.83 | |  |  |
| 74-0672 | chr11:775136 | G/C | PDDC1 | | nonsynonymous | p.S24W | | 1 | | | 1 | | 3.7 | |  |  |
| 74-0672 | chr5:137533923 | A/G | CDC23 | | nonsynonymous | p.I326T | | 0.62 | | | 0.119 | | 5.42 | |  |  |
| 74-0672 | **chr2:71650660** | **A/T** | **ZNF638** | | **nonsynonymous** | **p.D1339V** | | **0.33** | | | **0** | | **1.17** | |  |  |
| 74-0672 | chr11:33090247 | A/G | TCP11L1 | | nonsynonymous | p.K390R | | 0.08 | | | 0.001 | | 1.03 | |  |  |
| 74-0672 | chr6:10705011 | A/T | PAK1IP1 | | nonsynonymous | p.E225V | | 0.05 | | | 0.997 | | 5.66 | |  |  |
| 74-0672 | chr20:33879743 | T/C | FAM83C | | nonsynonymous | p.D122G | | 0.01 | | | 1 | | 4.7 | |  |  |
| 74-0672 | chr20:62187756 | T/C | C20orf195 | | nonsynonymous | p.L247P | | 0 | | | 0.999 | | 5.31 | |  |  |
| 74-0672 | chrX:102004930 | C/G | BHLHB9 | | nonsynonymous | p.T336S | | . | | | 0.346 | | 3.72 | |  |  |
| 74-0707 | chr1:29385338 | A/C | EPB41 | | nonsynonymous | p.T640P | | 0.33 | | | 0 | | -1.72 | |  |  |
| 74-0707 | **chr1:32196666** | **T/A** | **BAI2** | | **nonsynonymous** | **p.D1339V** | | **0.12** | | | **0.862** | | **3.82** | |  |  |
| 74-0752 | chr4:89772306 | A/G | FAM13A | | nonsynonymous | p.I291T | | 0.55 | | | 0.001 | | 3.25 | |  |  |
| 74-0752 | chr10:96740996 | C/T | CYP2C9 | | Stopgain | p.Q340X | | 0.52 | | | . | | 2.78 | |  |  |
| 74-0752 | chr17:35300106 | C/G | LHX1 | | nonsynonymous | p.P300R | | 0.22 | | | 1 | | 4.09 | |  |  |
| 74-0752 | chr4:100503235 | T/G | MTTP | | nonsynonymous | p.L79V | | 0.14 | | | 0.235 | | 3.07 | |  |  |
| 74-0752 | **chr9:80877854** | **A/C** | **CEP78** | | **nonsynonymous** | **p.K472T** | | **0.05** | | | **1** | | **4.37** | |  |  |
| 74-0752 | chr5:130771810 | G/T | RAPGEF6 | | nonsynonymous | p.T1337K | | 0.02 | | | 0.002 | | 5.02 | |  |  |
| 74-0752 | chr4:17782809 | C/T | FAM184B | | nonsynonymous | p.M38I | | 0.01 | | | 0.694 | | 4.58 | |  |  |
| 74-0752 | chr22:22893497 | G/C | PRAME | | nonsynonymous | p.S12R | | . | | | . | | -3.4 | |  |  |
| 74-0765 | chr10:1405594 | G/A | ADARB2 | | nonsynonymous | p.L236F | | 1 | | | 0 | | -0.315 | |  |  |
| 74-0765 | chr15:40269017 | C/G | EIF2AK4 | | nonsynonymous | p.H741D | | 1 | | | 0 | | 3.38 | |  |  |
| 74-0765 | chr15:45386852 | C/T | DUOX2 | | nonsynonymous | p.R1478Q | | 0.28 | | | 0.911 | | 5.68 | |  |  |
| 74-0765 | chr4:70723347 | C/A | SULT1E1 | | nonsynonymous | p.D6Y | | 0.22 | | | 0.001 | | 2.63 | |  |  |
| 74-0765 | chr6:17626137 | G/C | NUP153 | | nonsynonymous | p.T1226R | | 0.19 | | | 0.659 | | 4.83 | |  |  |
| 74-0765 | chr15:41384317 | C/G | INO80 | | nonsynonymous | p.E149Q | | 0.16 | | | 0.596 | | 5.37 | |  |  |
| 74-0765 | chr3:14862257 | T/C | FGD5 | | nonsynonymous | p.V560A | | 0.01 | | | 0.682 | | 1.2 | |  |  |
| 74-0765 | chr19:55606079 | G/T | PPP1R12C | | nonsynonymous | p.N513K | | 0.01 | | | 0.865 | | -0.763 | |  |  |
| 74-0765 | chr13:25487103 | T/A | CENPJ | | nonsynonymous | p.M21L | | 0 | | | 0.647 | | 5.28 | |  |  |
| 74-0765 | chr15:64275843 | C/A | DAPK2 | | nonsynonymous | p.R68L | | 0 | | | 0.866 | | 4.41 | |  |  |
| 74-0765 | chr10:96745800 | T/C | CYP2C9 | | nonsynonymous | p.I387T | | 0 | | | 0.995 | | 3.57 | |  |  |

Supplementary Table 3: Available phenotypic information for each subject from the exome-sequenced families.

| Family Id | SUBJECT ID | Age at Testing (years) | Age first phrases used (months) | Age first words used (months) | History of seizures | History of regression | Age of walking (months) | Age of symptom onset (parent report) | Diagnosis | Race | Sex | Aerbal Ability | Nonverbal IQ estimate | Verbal IQ estimate |
| --- | --- | --- | --- | --- | --- | --- | --- | --- | --- | --- | --- | --- | --- | --- |
| 152-HSC0079 | 152-HSC0079-3 | 5 | 18 | 12 | no | No | 12 | unknown | Spectrum | Other | male | unknown | 109 | unknown |
|  | 152-HSC0079-4 | 3 | 31 | 24 | no | No | 15 | 18 | Spectrum | Other | female | unknown | 113 | unknown |
|  | 152-HSC0079-8 | 4 | 30 | 15 | no | No | 12 | 27 | Autism | Other | male | unknown | 124 | unknown |
| 156-3925 | 156-3925-201 | 7 | 15 | 11 | no | No | 12 | 36 | Spectrum | White | male | fluent speech | unknown | unknown |
|  | 156-3925-351 | 11 | 60 | 48 | no | No | 30 | 12 | Autism | White | male | unknown | unknown | unknown |
| 60-1015 | 60-1015-002 | Unknown | unknown | unknown | unknown | unknown | unknown | 6 | Autism | White | female | unknown | unknown | unknown |
|  | 60-1015-023 | Unknown | unknown | unknown | unknown | unknown | unknown | 5 | Autism | White | male | unknown | unknown | unknown |
| 60-1049 | 60-1049-003 | Unknown | unknown | unknown | unknown | unknown | unknown | 24 | Autism | Other | male | unknown | unknown | unknown |
|  | 60-1049-023 | Unknown | unknown | unknown | unknown | unknown | unknown | 30 | Autism | Other | female | unknown | unknown | unknown |
|  | 60-1049-024 | Unknown | unknown | unknown | unknown | unknown | unknown | 18 | Autism | Other | male | unknown | unknown | unknown |
| 60-1057 | 60-1057-004 | Unknown | unknown | unknown | unknown | unknown | unknown | 15 | Autism | White | female | unknown | unknown | unknown |
|  | 60-1057-008 | Unknown | unknown | unknown | unknown | unknown | unknown | 12 | Autism | White | female | unknown | unknown | unknown |
| 60-2036 | 60-2036-003 | Unknown | unknown | unknown | unknown | unknown | unknown | 33 | Autism | White | male | unknown | unknown | unknown |
|  | 60-2036-008 | Unknown | unknown | unknown | unknown | unknown | unknown | 20 | Autism | White | male | unknown | unknown | unknown |
| 60-4031 | 60-4031-001 | Unknown | unknown | unknown | unknown | unknown | unknown | 24 | Autism | White | male | unknown | unknown | unknown |
|  | 60-4031-006 | Unknown | unknown | unknown | unknown | unknown | unknown | 24 | Autism | White | male | unknown | unknown | unknown |
| 72-0614 | 72-0614-04 | 9 | 30 | 12 | yes | yes | 11 | 18 | Autism | White | male | fluent speech | 75 | 50 |
|  | 72-0614-311 | 7 | 27 | 16 | no | No | 13 | 24 | Autism | White | male | fluent speech | 119 | 118 |
| 72-0745 | 72-0745-03 | 9 | 30 | 18 | no | No | 12 | 15 | Autism | White | male | fluent speech | 93 | 84 |
|  | 72-0745-301 | 7 | unknown but delayed | 54 | febrile seizures only | No | 12 | 12 | Autism | White | male | unknown | unknown | unknown |
| 72-1047 | 72-1047-301 | 11 | 48 | 43 | no | No | 11 | 22 | Autism | White | male | fluent speech | 107 | 118 |
|  | 72-1047-302 | 9 | 54 | 36 | no | No | 12 | 6 | Autism | White | male | fluent speech | 95 | 100 |
|  | 72-1047-312 | 10 | 54 | 48 | no | No | 10 | 22 | Autism | White | male | fluent speech | 80 | 62 |
| 72-1397 | 72-1397-302 | 7 | 48 | 42 | no | No | 13 | 18 | Autism | Other | male | fluent speech | 125 | 97 |
|  | 72-1397-313 | 7 | 42 | 23 | no | No | 15 | 12 | Autism | Other | male | fluent speech | 119 | 90 |
| 72-1921 | 72-1921-305 | 3 | not attained at time of testing | 15 | yes | yes | 16 | 1 | Autism | Other | male | single words/nonverbal | unknown | unknown |
|  | 72-1921-311 | 3 | 28 | 12 | no | no | 12 | 1 | Spectrum | Other | female | phrase speech | 93 | 112 |
| 74-0081 | 74-0081-03 | 7 | 72 | 24 | no | yes | 15 | 18 | Spectrum | Other | male | fluent speech | 97 | unknown |
|  | 74-0081-04 | 6 | 47 | 45 | no | No | 14 | 9 | Autism | Other | male | fluent speech | 100 | unknown |
|  | 74-0081-303 | 6 | 36 | 24 | no | No | 14 | 24 | Autism | Other | male | fluent speech | 85 | 86 |
| 74-0180 | 74-0180-03 | 6 | 54 | 36 | no | yes | 12 | 30 | Autism | Other | male | unknown | 108 | 80 |
|  | 74-0180-04 | 4 | 33 | 33 | no | yes | 11 | 18 | Autism | Other | male | unknown | unknown | 59 |
|  | 74-0180-10 | 13 | 14 | 10 | yes | yes | 12 | 16 | Autism | Other | male | unknown | unknown | unknown |
| 74-0264 | 74-0264-04 | 7 | 75 | 69 | no | No | 12 | 10 | Autism | White | male | phrase speech | unknown | 31 |
|  | 74-0264-05 | 6 | 33 | 23 | no | No | 12 | 10 | Autism | White | female | fluent speech | 127 | 104 |
|  | 74-0264-12 | 10 | 48 | 36 | no | No | 11 | 24 | Autism | White | male | unknown | unknown | unknown |
| 74-0358 | 74-0358-10 | 6 | 27 | 24 | no | yes | 10 | 30 | Autism | Other | male | single words/nonverbal | unknown | 34 |
|  | 74-0358-13 | 5 | 45 | 42 | febrile seizures only | yes | 10 | 22 | Spectrum | Other | female | phrase speech | unknown | 86 |
| 74-0450 | 74-0450-04 | 4 | 42 | 12 | no | yes | 11 | 16 | Autism | Other | male | phrase speech | 108 | 80 |
|  | 74-0450-10 | 10 | 22 | 15 | no | No | 9 | 36 | Spectrum | Other | male | fluent speech | 125 | 141 |
| 74-0455 | 74-0455-10 | 7 | 28 | 16 | no | no | 12 | 24 | Spectrum | Other | male | fluent speech | 131 | 114 |
|  | 74-0455-12 | 8 | 50 | 48 | no | No | 13 | 19 | Autism | Other | female | phrase speech | 80 | unknown |
|  | 74-0455-14 | 5 | 42 | 20 | no | No | 13 | 12 | Autism | Other | male | phrase speech | unknown | 84 |
| 74-0481 | 74-0481-03 | 12 | 61 | 58 | unknown | unknown | unknown | 18 | Autism | White | male | phrase speech | 100 | 56 |
|  | 74-0481-312 | 17 | 72 | 54 | no | No | 8 | 24 | Autism | White | male | fluent speech | 78 | 76 |
| 74-0672 | 74-0672-08 | 3 | not attained at time of testing | not attained at time of testing | no | No | 12 | 8 | Autism | White | male | single words/nonverbal | unknown | unknown |
|  | 74-0672-09 | 3 | 25 | 15 | no | yes | 18 | 15 | Autism | White | female | phrase speech | 95 | 100 |
| 74-0707 | 74-0707-03 | 10 | 19 | 14 | no | No | 12 | 36 | Spectrum | White | male | fluent speech | unknown | 125 |
|  | 74-0707-05 | 4 | 42 | 15 | no | yes | 13 | 30 | Autism | White | male | phrase speech | unknown | 90 |
|  | 74-0707-10 | 19 | unknown but delayed | unknown but on time | no | No | 13 | 24 | Spectrum | White | male | fluent speech | 75 | 97 |
| 74-0752 | 74-0752-07 | 5 | 54 | 54 | no | yes | 12 | 18 | Spectrum | Hispanic | female | single words/nonverbal | 100 | 76 |
|  | 74-0752-10 | 4 | not attained at time of testing | not attained at time of testing | no | No | 13 | 24 | Autism | Hispanic | male | single words/nonverbal | unknown | 49 |
| 74-0765 | 74-0765-08 | 8 | 72 | 60 | no | No | 12 | 8 | Autism | Other | male | fluent speech | 85 | 80 |
|  | 74-0765-09 | 4 | 36 | 16 | no | yes | 15 | 18 | Spectrum | Other | female | single words/nonverbal | 88 | 86 |

Supplementary Table 4: Overrepresentation Enrichment Analysis using Gene Ontology (GO) database for the genes with VOIs identified in exome sequencing. C: Number of reference gene in the category; O: Number of observed genes in the category; E: Expected number of genes in the category; R: Ratio of enrichment; P value: p value from hypergeometric test; FDR: FDR using Benjamini-Hochberg Procedure.

| GO Category | GO Term | C | O | E | R | P Value | FDR | Observed VOI Genes |
| --- | --- | --- | --- | --- | --- | --- | --- | --- |
| Biological Process | cell projection organization (GO:0030030) | 1268 | 23 | 8.66 | 2.66 | 1.26E-05 | 0.0039 | CEP250;FGD5;TTC21A;FSTL4;LHX1;MAP1B;MAP2;EIF2AK4;NCK1;MINK1;TRPV2;DCDC2;RAPGEF6;PLXNB1;CENPJ;PRKCQ;IFT46;LRFN2;KLHL1;BHLHB9;SSH2;SCARF1;CEP41 |
|  | neuron projection morphogenesis (GO:0048812) | 552 | 12 | 3.77 | 3.18 | 3.86E-04 | 0.0321 | FSTL4;LHX1;MAP1B;MAP2;EIF2AK4;MINK1;TRPV2;PLXNB1;PRKCQ;LRFN2;BHLHB9;SSH2 |
|  | neuron projection development (GO:0031175) | 811 | 15 | 5.54 | 2.71 | 4.02E-04 | 0.0321 | FSTL4;LHX1;MAP1B;MAP2;EIF2AK4;NCK1;MINK1;TRPV2;PLXNB1;PRKCQ;LRFN2;KLHL1;BHLHB9;SSH2;SCARF1 |
|  | cell projection morphogenesis (GO:0048858) | 569 | 12 | 3.88 | 3.09 | 5.06E-04 | 0.0321 | FSTL4;LHX1;MAP1B;MAP2;EIF2AK4;MINK1;TRPV2;PLXNB1;PRKCQ;LRFN2;BHLHB9;SSH2 |
|  | cell morphogenesis involved in neuron differentiation (GO:0048667) | 506 | 11 | 3.45 | 3.18 | 6.82E-04 | 0.0321 | FSTL4;LHX1;MAP1B;MAP2;MINK1;TRPV2;PLXNB1;PRKCQ;LRFN2;BHLHB9;SSH2 |
|  | cell part morphogenesis (GO:0032990) | 589 | 12 | 4.02 | 2.98 | 6.87E-04 | 0.0321 | FSTL4;LHX1;MAP1B;MAP2;EIF2AK4;MINK1;TRPV2;PLXNB1;PRKCQ;LRFN2;BHLHB9;SSH2 |
|  | neuron development (GO:0048666) | 951 | 16 | 6.49 | 2.46 | 7.17E-04 | 0.0321 | FSTL4;LHX1;MAP1B;MAP2;EIF2AK4;NCK1;MINK1;TRPV2;PLXNB1;PRKCQ;LRFN2;KLHL1;WNK1;BHLHB9;SSH2;SCARF1 |
| Cellular Component | cytoskeletal part (GO:0044430) | 1489 | 17 | 6.59 | 2.58 | 2.22E-04 | 0.0063 | TUBGCP2;CEP250;NEK10;EPB41;FBXO5;KRTAP19-3;KRT86;MAP1B;MAP2;DCDC2;INO80;TCP11L1;CENPJ;IFT46;CEP78;CEP41;SNPH |
|  | cell junction (GO:0030054) | 1357 | 16 | 6.00 | 2.67 | 2.45E-04 | 0.0063 | CHRM4;EPB41;FGFR4;MPRIP;ARHGEF16;PCLO;ITGB6;MAP1B;NCK1;MINK1;DUOX2;PLEKHG5;LRFN2;CAT;CEP41;SNPH |
|  | plasma membrane region (GO:0098590) | 923 | 12 | 4.08 | 2.94 | 6.98E-04 | 0.0098 | CFTR;CHRM4;FGD5;WWC1;IQSEC3;MINK1;DUOX2;RAPGEF6;SLC22A11;LRFN2;HYAL2;SNPH |
|  | microtubule cytoskeleton (GO:0015630) | 1069 | 13 | 4.73 | 2.75 | 7.66E-04 | 0.0098 | TUBGCP2;CEP250;NEK10;FBXO5;MAP1B;MAP2;INO80;TCP11L1;CENPJ;IFT46;CEP78;CEP41;SNPH |
|  | polymeric cytoskeletal fiber (GO:0099513) | 669 | 9 | 2.96 | 3.04 | 2.71E-03 | 0.0226 | TUBGCP2;KRTAP19-3;KRT86;MAP1B;MAP2;INO80;TCP11L1;CENPJ;SNPH |
|  | supramolecular fiber (GO:0099512) | 696 | 9 | 3.08 | 2.92 | 3.53E-03 | 0.0226 | TUBGCP2;KRTAP19-3;KRT86;MAP1B;MAP2;INO80;TCP11L1;CENPJ;SNPH |
|  | supramolecular polymer (GO:0099081) | 703 | 9 | 3.11 | 2.89 | 3.77E-03 | 0.0226 | TUBGCP2;KRTAP19-3;KRT86;MAP1B;MAP2;INO80;TCP11L1;CENPJ;SNPH |
|  | supramolecular complex (GO:0099080) | 704 | 9 | 3.11 | 2.89 | 3.80E-03 | 0.0226 | TUBGCP2;KRTAP19-3;KRT86;MAP1B;MAP2;INO80;TCP11L1;CENPJ;SNPH |
|  | cell surface (GO:0009986) | 709 | 9 | 3.14 | 2.87 | 3.98E-03 | 0.0226 | CFTR;GPRC6A;SCUBE3;ITGB6;TRPV2;SLC22A11;LRFN2;HYAL2;CEP41 |
|  | synapse part (GO:0044456) | 626 | 8 | 2.77 | 2.89 | 6.35E-03 | 0.0324 | CHRM4;EPB41;PCLO;MAP1B;IQSEC3;MINK1;LRFN2;SNPH |
| Molecular Function | kinase binding (GO:0019900) | 587 | 13 | 3.45 | 3.77 | 3.81E-05 | 0.0016 | CEP250;FAM83C;EPHA1;WWC1;FBXO5;CNPPD1;ARHGEF16;NCK1;NFATC1;PPP1R12C;CENPJ;WNK1;HYAL2 |
|  | protein kinase binding (GO:0019901) | 522 | 12 | 3.07 | 3.91 | 5.42E-05 | 0.0016 | CEP250;FAM83C;EPHA1;FBXO5;CNPPD1;ARHGEF16;NCK1;NFATC1;PPP1R12C;CENPJ;WNK1;HYAL2 |

Supplementary Table 5: Overrepresentation Enrichment Analysis using Gene Ontology (GO) database for the 111 ASD genes downloaded from SFARI database. C: Number of reference genes in the category; O: Number of observed genes in the category; E: Expected number of genes in the category; R: Ratio of enrichment; P value: p value from hypergeometric test; FDR: FDR using Benjamini-Hochberg Procedure.

Supplementary Table 6: Rare nonsynonymous variants identified by MIP sequencing. **SIFT**(Sorting Intolerant From Tolerant) predicts impact of amino acid substitutions based on the degree of conservation in sequence alignments derived from closely related sequences. Scores <0.05 are considered deleterious. **PolyPhen-2**(Polymorphism Phenotyping v2) predicts impact of a variant on the structure and function of a human protein using eight sequence-based and three structure-based predictive features. Scores >0.95 are considered probably damaging. **GERP** (Genomic Evolutionary Rate Profiling) identifies functional constraint of a sequence variant by quantifying substitution deficits in multiple alignments. Substitution deficits represent a natural measure of constraint that reflects the strength of past purifying selection. Higher GERP scores are more deleterious. **ExAC NFN** is the frequency of the variants in Non-Finnish European Population of ExAC database.

Supplementary Table 7: Gene based variant-burden association study for selected candidate genes in 1004 familial cases and 1127 unscreened controls. **Case**: Number of ASD cases with the variant / Number of ASD cases without the variant. **Control**: Number of controls with the variant / Number of controls without the variant.

| Gene | Case | Control | P value^a^ |
| --- | --- | --- | --- |
| *BAI2* | 38/966 | 48/1079 | 0.7543 |
| *CEP41* | **18/986** | **2/1125** | **6.185e-05*** |
| *CEP78* | 19/985 | 23/1104 | 0.6569 |
| *CEP250* | 36/968 | 34/1093 | 0.2611 |
| *DCDC2* | 5/999 | 8/1119 | 0.8177 |
| *NFATC1* | 6/998 | 10/1117 | 0.8486 |
| *NTAN1* | 5/999 | 5/1122 | 0.5501 |
| *SCN10A* | 74/930 | 83/1044 | 0.5304 |
| *SH3BP4* | 27/977 | 32/1095 | 0.6356 |
| *SNPH* | 11/993 | 8/1119 | 0.2345 |
| *SPATA3* | 6/998 | 6/1121 | 0.5325 |
| *TMEM82* | 21/983 | 16/1111 | 0.1491 |
| *ZNF638* | 9/995 | 15/1112 | 0.8786 |

^a^p-value was determined by one sided Fisher Exact test, significant p-value was marked in bold

*Significant after Bonferroni correction (corrected p value = 9.2866e-04)

Supplementary Table 8: Available phenotypic information for the cases with CEP41 rare nonsynonymous variants identified by MIP sequencing.

| Subject Id | Gender | Age (years) | VIQ | NVIQ | Other | First Symptoms (months) | Word Delay | Phrase Delay | Language | Regression | Seizures | Walking (months) | Gait |
| --- | --- | --- | --- | --- | --- | --- | --- | --- | --- | --- | --- | --- | --- |
| 3304 | female | 13 | 94 | 67 | tics, anxiety, OCD | 2 | Yes | Yes | fluent | No | No | unknown | unknown |
| 4904 | female | 13 | 64 | 97 | ODD, OCD, depression, anxious symptoms | 9 | No | Yes | fluent | Yes | No | unknown | unknown |
| 20703 | male | 6 | 108 | 106 | subthreshold depression, anxiety symptoms, | 5 | No | No | fluent | No | No | unknown | unknown |
| 152-MM0063-3 | female | 3 | unknown | 71 | unknown | 9 | Yes | Yes | minimally verbal | No | unknown | 12 | unknown |
| 154-19703 | female | 18 | 81 | 64 | unknown | 12 | No | No | fluent | No | unknown | unknown | unknown |
| 60-1106-004 | Male | 3 | unknown | 44 | unknown | 12 | unknown | unknown | unknown | unknown | unknown | unknown | unknown |
| 63-8701 | Male | 4 | unknown | unknown | unknown | 14 | unknown | unknown | unknown | unknown | unknown | unknown | unknown |
| 72-0907-302 | Male | 8 | 85 | 105 | unknown | 18 | No | No | fluent | No | No | 12 | normal |
| 72-1411-302 | Male | 2 | unknown | unknown | unknown | 1 | Yes | Yes | minimally verbal | No | No | 16 | normal |
| 72-1424-304 | Male | 6 | 102 | 103 | unknown | 24 | No | no | fluent | No | No | 13 | normal |
| 72-1510-302 | Male | 5 | unknown | unknown | unknown | 12 | Yes | Yes | minimally verbal | No | No | 13 | atypical gait |
| 74-0183-03 | Male | 7 | unknown | 119 | unknown | 12 | No | Yes | fluent | No | No | 14 | normal |
| 74-0196-05 | Male | 4 | unknown | unknown | unknown | 12 | Yes | Yes | phrase speech | No | No | 9 | atypical gait |
| 74-0626-03 | Male | 8 | 73 | 97 | unknown | 1 | Yes | Yes | phrase speech | No | No | 30 | atypical gait |

Supplementary Figures:


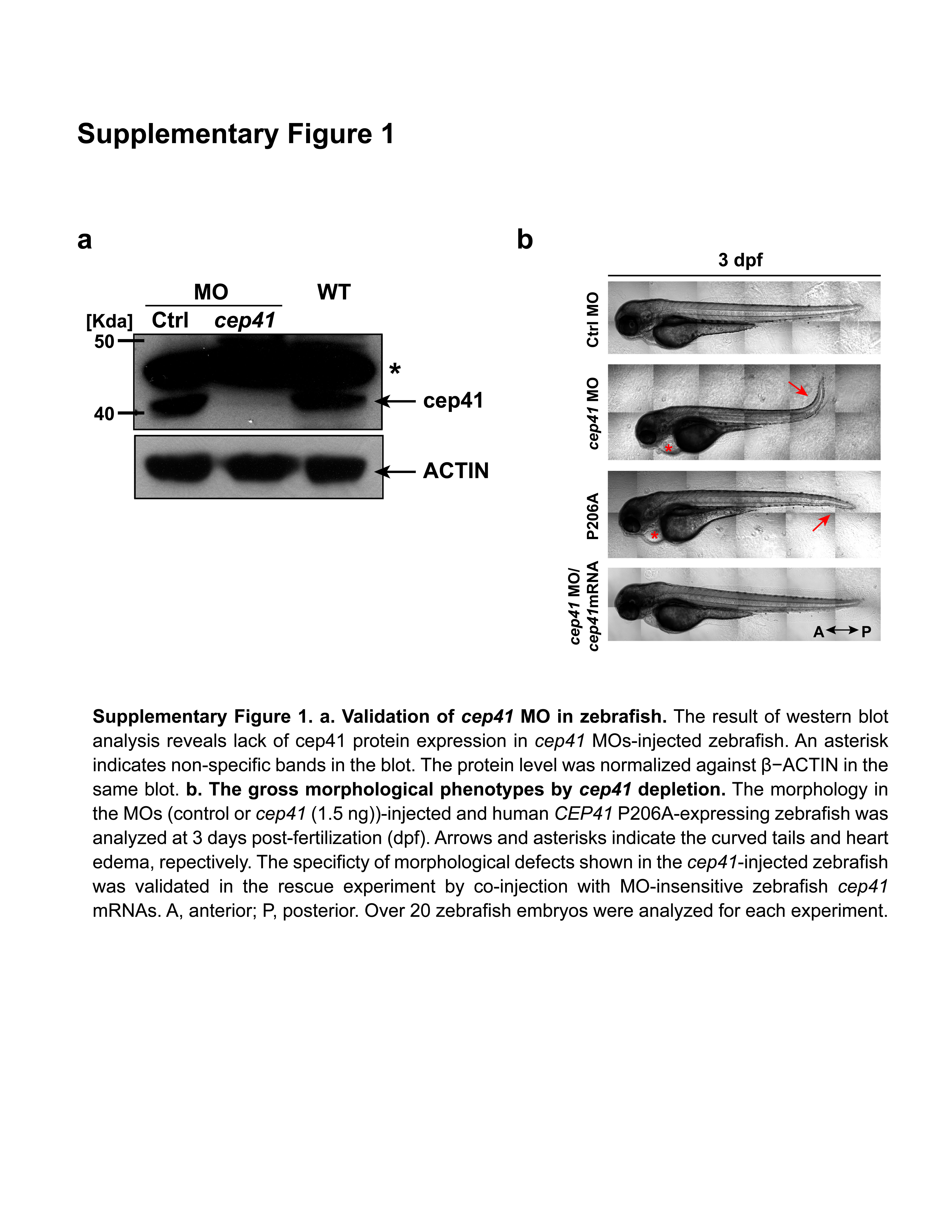


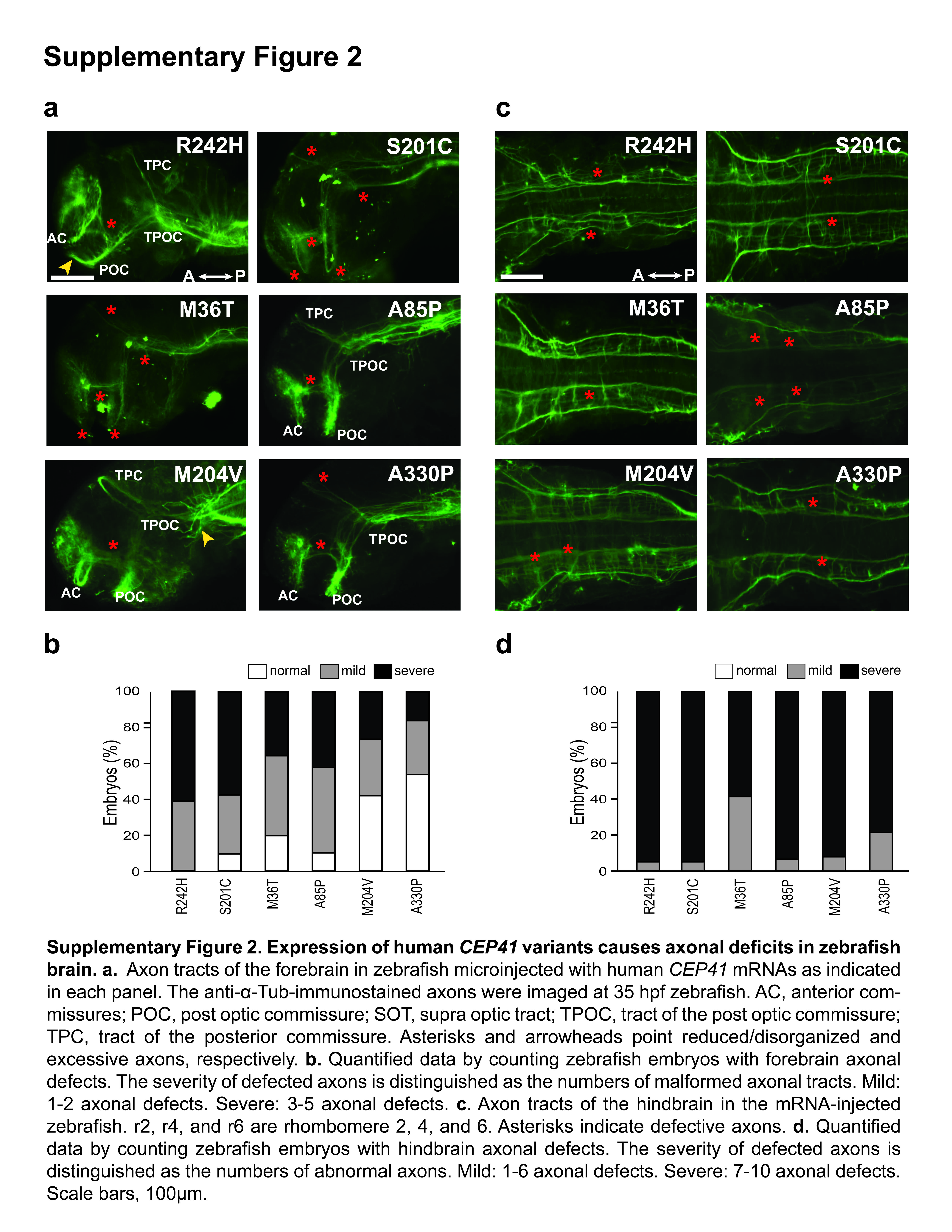


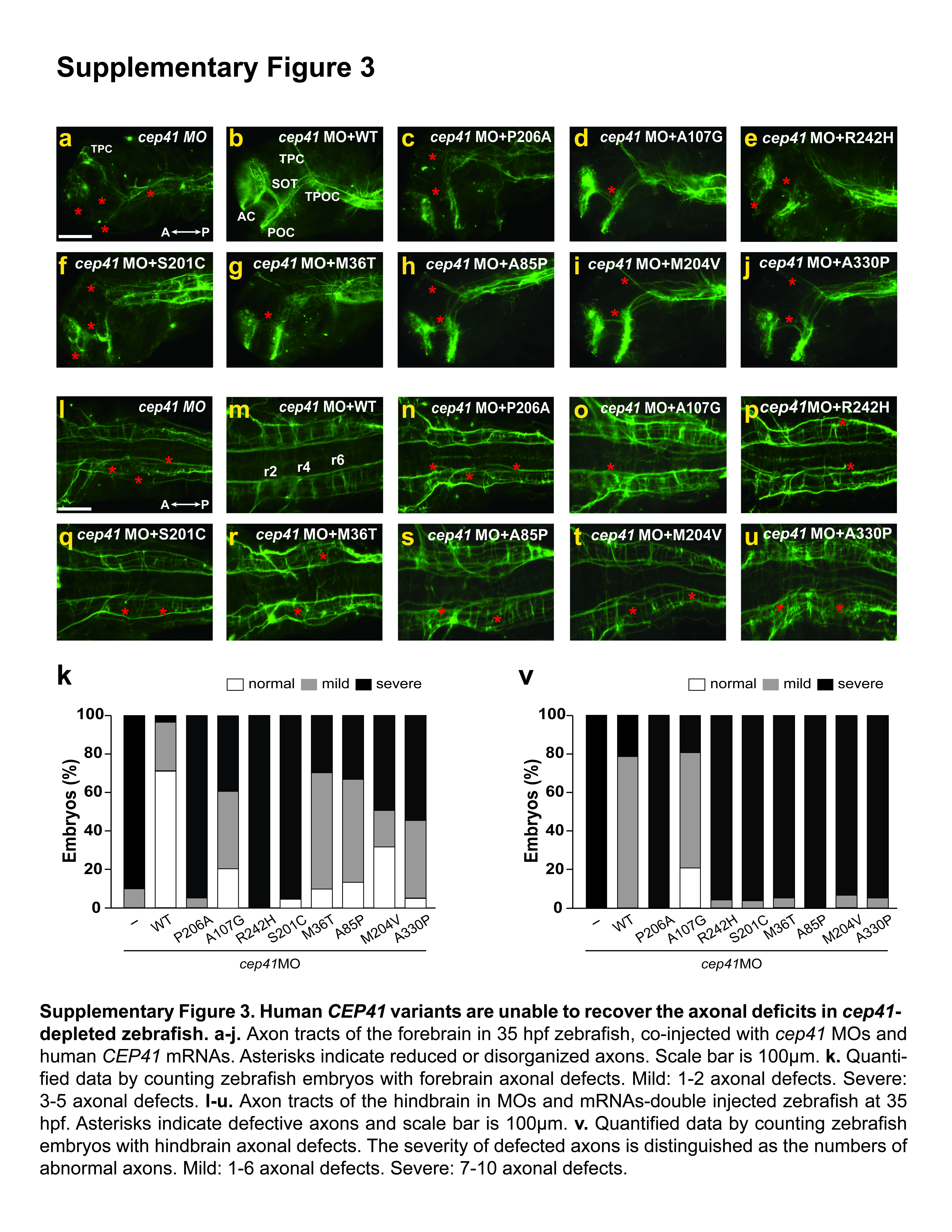


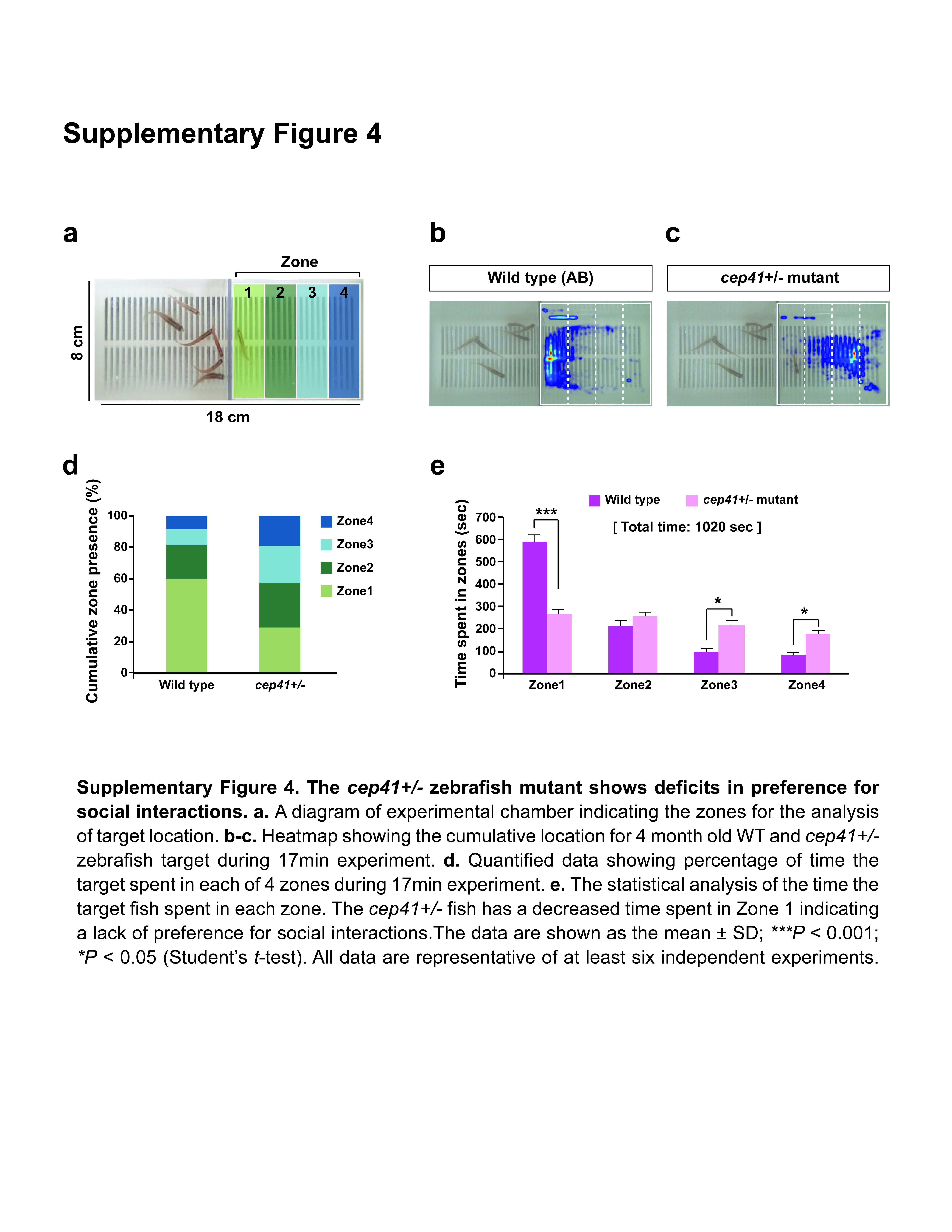


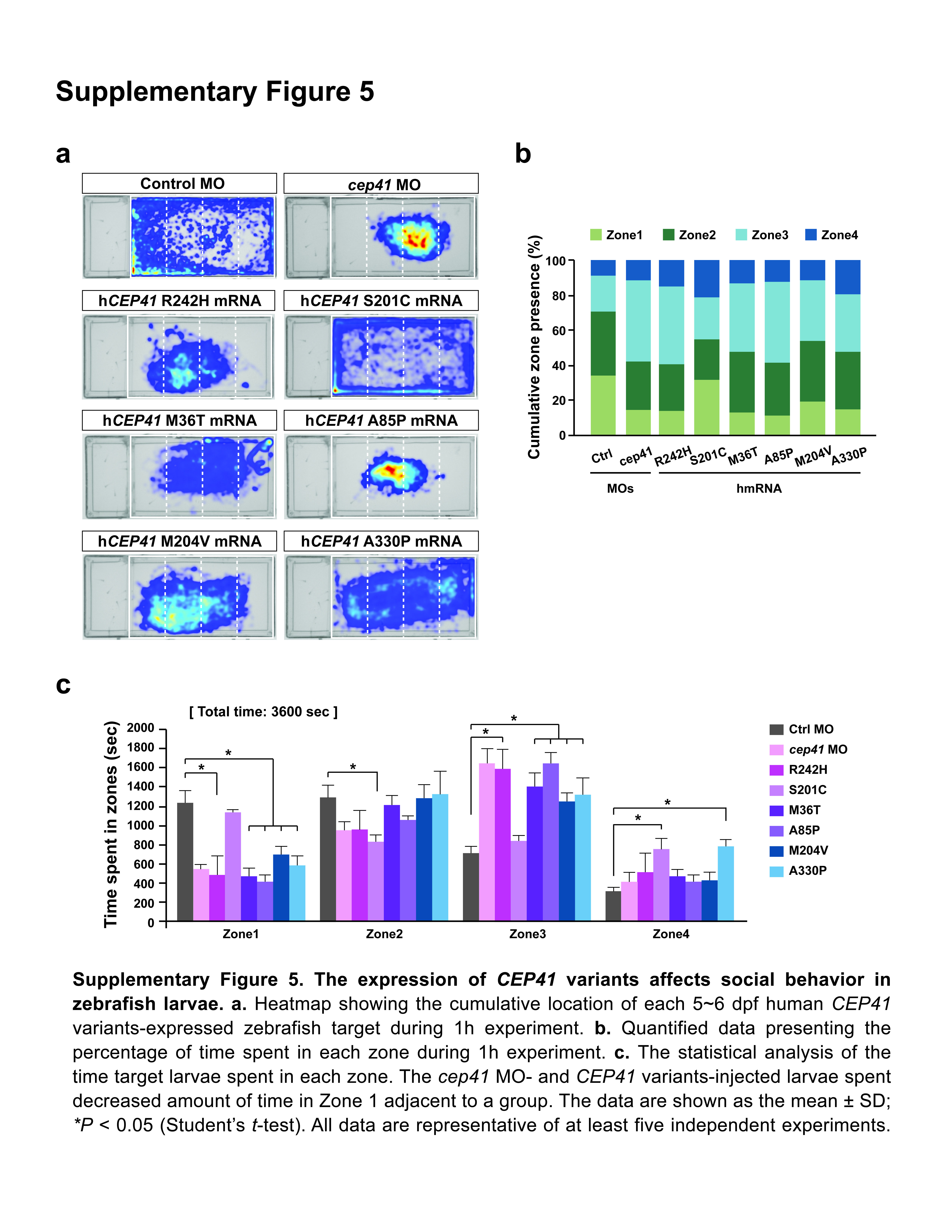


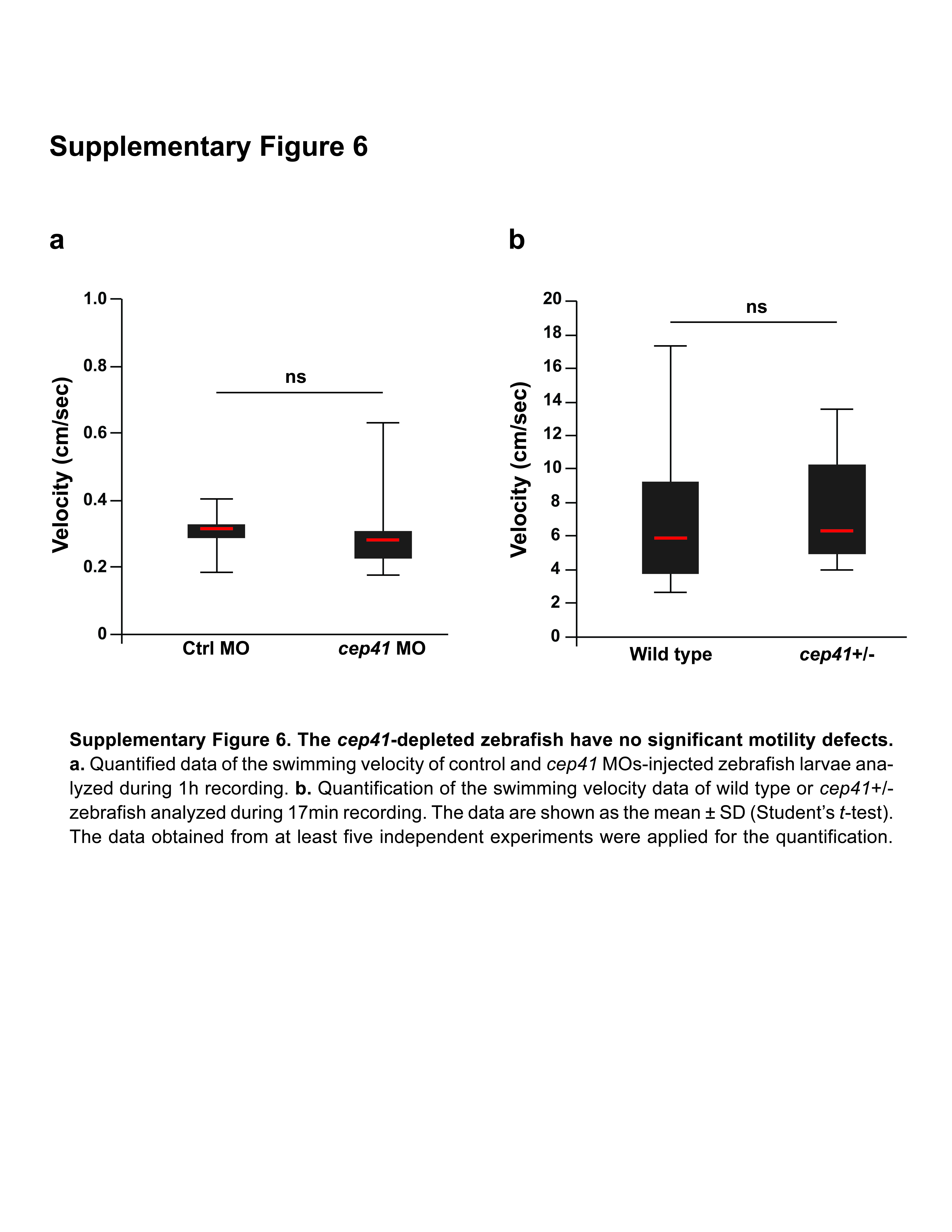


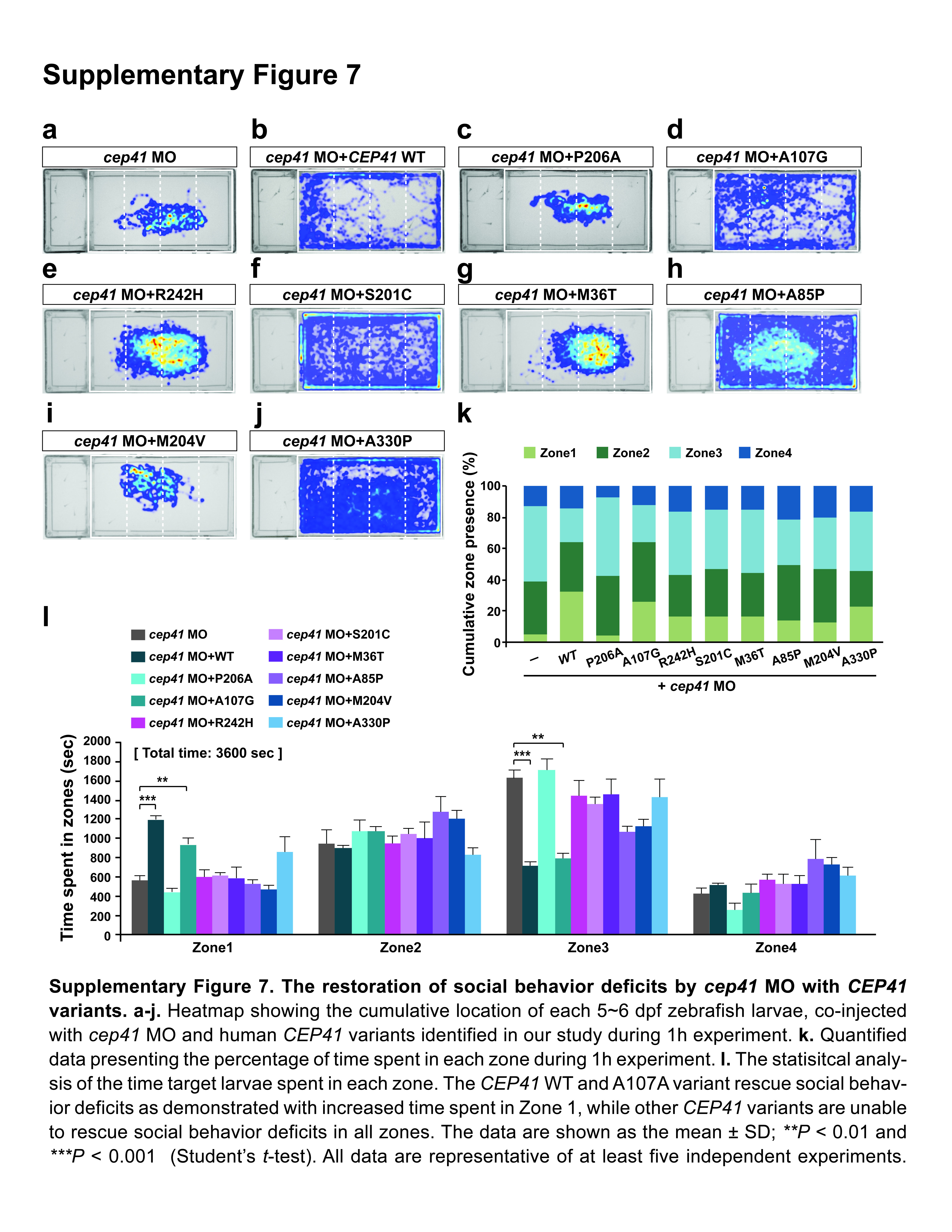

Supplement: Supplementary file 1 — Supplementary Information [file 41398_2018_343_MOESM1_ESM.docx]
